# Supplementary material for: A human antibody against pathologic IAPP aggregates protects beta cells in type 2 diabetes models
Source: Nat Commun. 2023 Oct 9;14:6294. doi: 10.1038/s41467-023-41986-0 (PMC10562398; doi:10.1038/s41467-023-41986-0)
Supplement: Supplementary file 1 — Supplementary Information [file 41467_2023_41986_MOESM1_ESM.pdf]

Supplementary Information

A human antibody against pathologic IAPP aggregates protects beta cells in type 2 diabetes models

Fabian Wirth[#1], Fabrice D. Heitz[#1], Christine Seeger[1], Ioana Combaluzier[1], Karin Breu[1], Heather C. Denroche[3], Julien Thevenet[4], Melania Osto[5], Paolo Arosio[6], Julie Kerr-Conte[4], C. Bruce Verchere[3], François Pattou[4], Thomas A. Lutz[5], Marc Y. Donath[7], Christoph Hock[1,2], Roger M. Nitsch[1,2], Jan Grimm[1]*

[1] Neurimmune AG, Wagistrasse 18, 8952 Schlieren, Switzerland

[2] Institute for Regenerative Medicine-IREM, University of Zürich, Wagistrasse 12, 8952 Schlieren, Switzerland

[3] BC Children's Hospital Research Institute and Centre for Molecular Medicine and Therapeutics, Departments of Surgery and Pathology & Laboratory Medicine, University of British Columbia, A4-151 950 W 28 Ave, Vancouver, Canada

[4] Univ-Lille, Inserm, CHU Lille, U1190 - EGID, F-59000 Lille, France

[5] Institute of Veterinary Physiology, Vetsuisse Faculty of the University of Zürich, Winterthurerstrasse 260, 8057 Zürich, Switzerland

[6] Institute for Chemical and Bioengineering, ETH Zürich, Vladimir-Prelog-Weg 1-5/10, 8093 Zürich, Switzerland

[7] Clinic for Endocrinology, Diabetes & Metabolism, and Department of Biomedicine, University Hospital Basel, Hebelstrasse 20, 4031 Basel, Switzerland

[#] Contributed equally

* Corresponding author: J. Grimm: jan.grimm@neurimmune.com

This PDF file includes:

Supplementary Figures 1 to 17

Supplementary Tables 1 to 3

Supplementary Methods

Supplementary References

Supplementary Figures

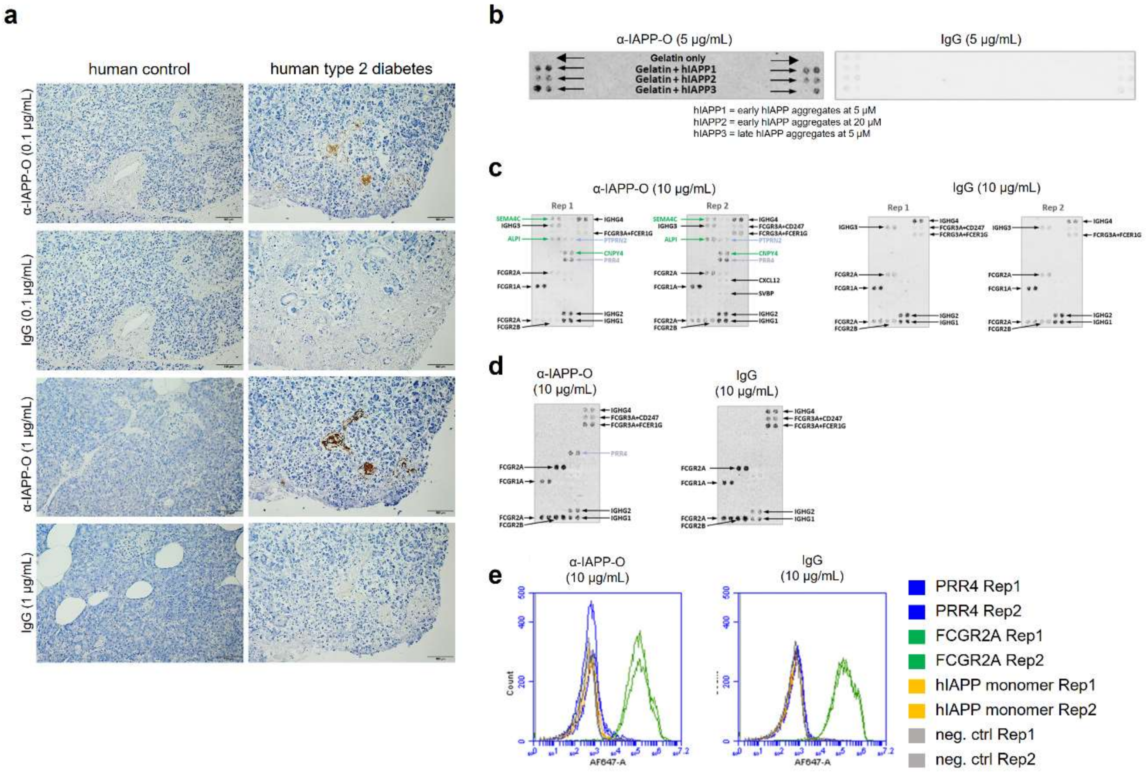

Supplementary Figure 1 (**a**) Representative bright-field images of pancreas sections from a human type-2 diabetes (T2D) and a non-diabetic control subject stained with FITC-labelled human α-IAPP-O (α-IAPP-O) or FITC-labelled human isotype control (IgG) antibodies at 0.1 and 1 µg/ml (corresponding to 0.67 and 6.7 nM) followed by an α -FITC rabbit polyclonal antibody combined with Bond Polymer Refine Detection Kit. Scale bars: 100 µm. (**b-e**) Off-target binding screening by microarray technology. (**b**) Pre-screen analysis: binding of α-IAPP-O and IgG to non-transfected HEK293 cells (gelatin only), and to slides spotted with different hIAPP aggregate preparations (Gelatin+hIAPP1-3). Antibodies were added to live cells before fixation. (**c**) Confirmation analysis upon identification of 43 hits derived from a library screen assessing binding of α-IAPP-O towards human HEK293 cells individually expressing 5868 full-length human plasma membrane proteins and cell surface-tethered human secreted proteins and 371 human heterodimers before fixation. Each library hit was re-expressed, along with control receptors and re-tested with 10 µg/mL α-IAPP-O and IgG. Upon pruning of non-reproducible and non-specific signals, 5 interactions (SEMA4C, ALPI, PTPRN2, CNPY4 and PRR4) characterized as potentially specific for α-IAPP-O on live cell microarrays before fixation remained. (**d**) Follow-up α-IAPP-O binding experiment on live cells with no post-fixation showed that 4 out of 5 interactions (SEMA4C, ALPI, PTPRN2, CNPY4) could not be replicated while PRR4 still remained positive, presumably being non-specific though as signal was also observed for IgG although to lesser extent (**e**) The interaction with PRR4 further investigated by flow cytometry on live cells revealed no binding signals with α-IAPP-O. Also, no interaction could be seen between α-IAPP-O and native hIAPP monomer displayed on HEK293 cells.

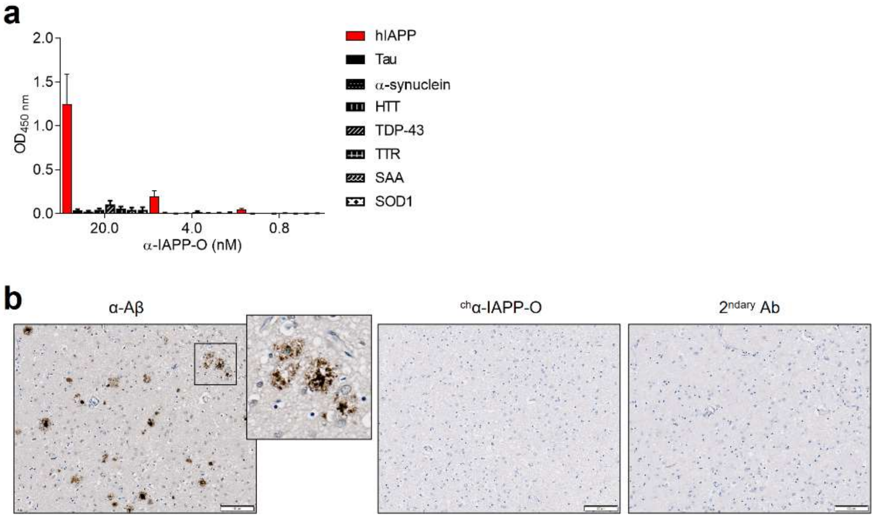

Supplementary Figure 2 Absence of α-IAPP-O cross-reactivity to unrelated amyloidogenic and misfolded proteins. (**a**) Binding of α-IAPP-O to human IAPP (hIAPP) aggregates, Tau, α-synuclein, huntingtin (HTT), TAR DNA-binding protein 43 (TDP-43), transthyretin (TTR), serum amyloid A (SAA) and superoxide dismutase 1 (SOD1) assessed by direct ELISA. OD_{450nm}, optical density at 450 nm. Data are means ± s.e.m. (**b**) Representative bright-field images of brain tissue sections from a patient with Alzheimer's disease showing Aβ plaques detected using an anti-Aβ antibody (in brown) and stained with mouse ^{ch}α-IAPP-O. Anti-mouse secondary antibody was used as control (2^{ndary} Ab). Scale bar: 100 μm.

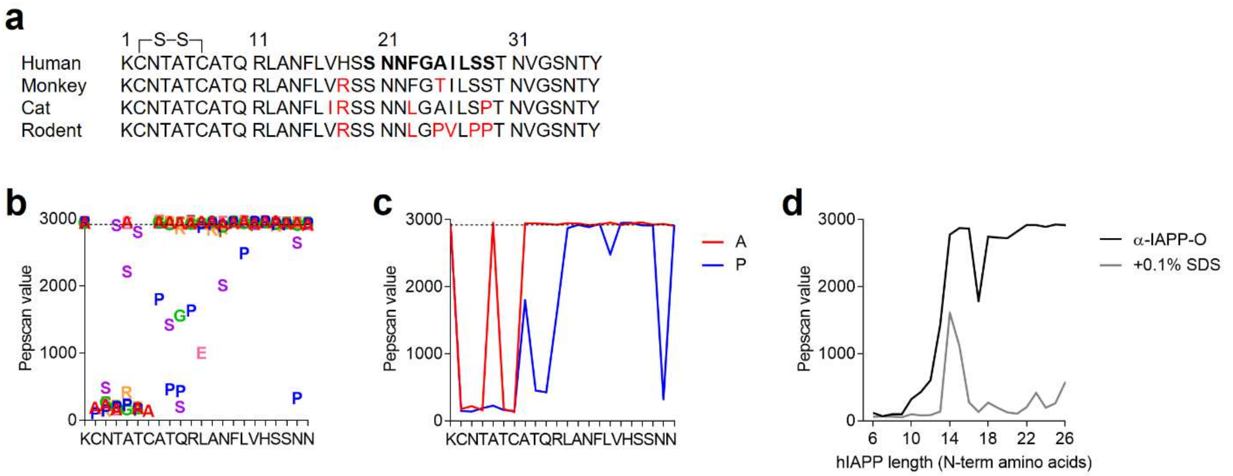

Supplementary Figure 3 α-IAPP-O binding to a conserved N-terminal epitope is strongly influenced by IAPP structure. (**a**) Comparison of human, rhesus monkey, cat, and rodent IAPP peptide sequences. Amino acid residues that differ from the human IAPP sequence are indicated in red and the human amyloidogenic region (20-29) essential for amyloid formation is represented in bold. A disulfide bond is formed between cysteine residues 2 and 7. Sequences are taken from[1]. (**b-d**) Epitope mapping of α-IAPP-O on 3D-structured hIAPP peptides showing critical roles for amino acids 2 to 7 at the N-terminus. Representation of α-IAPP-O binding to 22-mer peptides carrying single mutations indicated by the letter code of the amino acid at the position where the substitution is made (**b**), and contribution of alanine (A, red line) and proline (P, blue line) substitution that disrupt the peptide structure (**c**). The dashed line represents the mean signal obtained for the base peptide. Epitope recognition is enhanced for longer peptides (black line) and is impaired by C-terminal truncation and denaturation using 0.1% sodium dodecyl sulfate (SDS, grey line) (**d**).

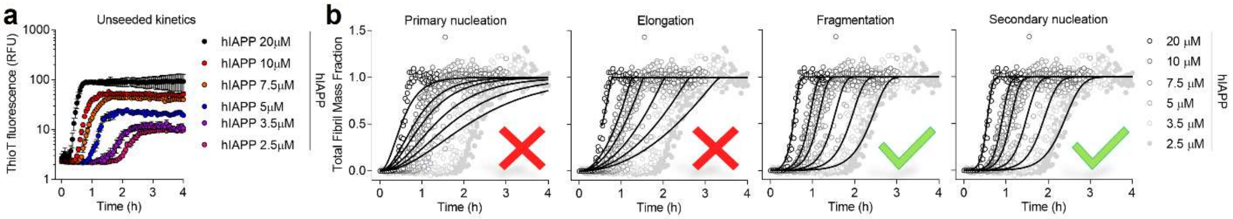

c

Dominant molecular mechanisms	Global fit	k_+k_n ($M^{-n_c}.min^{-2}x10^6$)	k_+k_2 ($M^{-n_2-1}.min^{-2}$)	k_+k_- ($M^{-1}.min^{-2}$)	n_c	n_2	Mean residual error
Primary nucleation	-						
Elongation	-						
Fragmentation	+	6.91	NA	563	2.29	NA	0.00999
Secondary nucleation	+	0.152	7.10x10^3	NA	2.01	0.185	0.00976

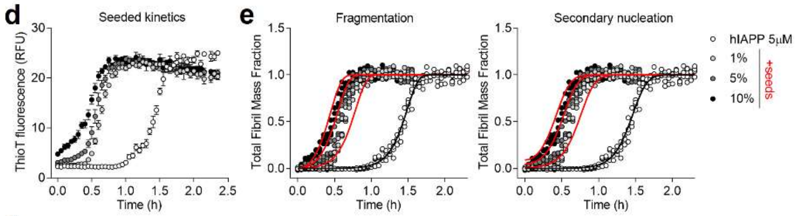

f

Dominant molecular mechanisms	Fitted parameters						Fixed parameters	
	Primary nucleation, k_n ($M^{-n_c+1}.min^{-1}x10^8$)	Elongation, k_+ ($M^{-1}.min^{-1}x10^{-3}$)	Secondary nucleation, k_2 ($M^{-n_2}.min^{-1}x10^5$)	Fragmentation, k_- ($min^{-1}x10^4$)	Depolymerisation, k_{off} ($min^{-1}x10^{-18}$)	Mean residual error	n_c	n_2
Fragmentation	9.09	86.1	NA	1.46	8.81	0.00638	2.29	NA
Secondary nucleation	1.6	14.7	8.76	NA	NA	0.00692	2.01	0.185

Supplementary Figure 4 Identification of the microscopic mechanisms underlying hIAPP aggregation. (**a**) Aggregation profile of hIAPP as a function of the initial peptide concentration monitored by thioflavin-T (ThioT) fluorescence intensity over time. Data are means ± s.e.m. from four replicates. (**b**) Kinetic analysis with global fit compatible with both fragmentation-dominated and secondary nucleation-dominated mechanisms. (**c**) Resulting product of elongation and primary nucleation rate constants (k_+k_n), elongation and secondary nucleation rate constants (k_+k_2), and elongation and fragmentation rate constants (k_+k_-). Shading in grey indicates poor global fit. n_c, primary nucleus size; n_2, secondary nucleus size; NA, not applicable. (**d**) Aggregation kinetics of hIAPP (5 μM) in the presence of 1, 5 and 10% seeds (pre-formed hIAPP fibrils). Experimental thioflavin-T (ThioT) fluorescence data are means ± s.e.m. from four replicates. (**e**,**f**) Kinetic analysis of data shown in **d** assuming fragmentation and secondary nucleation-dominated fibril formation (**e**) with corresponding kinetic rates upon fitting (**f**).

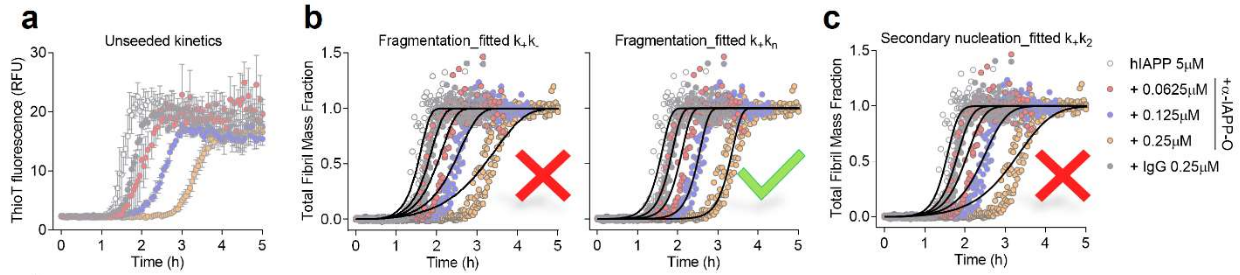

d

		Fragmentation_fitted parameters		Secondary nucleation_fitted parameters	
		k_+k_- ($M^{-1}.min^{-2}$)	k_+k_n ($M^{-n_c}.min^{-2}x10^6$)	k_+k_2 ($M^{-n_2-1}.min^{-2}$)	k_+k_n ($M^{-n_c}.min^{-2}x10^6$)
hIAPP 5μM	+vehicle		5.209163		0.092498
	+α-IAPP-O 0.0625μM		0.559938		0.007388
	+α-IAPP-O 0.125μM		0.076290		0.000745
	+α-IAPP-O 0.25μM		0.002015		0.000011
	+IgG 0.25μM		1.651353		0.024992

Supplementary Figure 5 α-IAPP-O concentration-dependently inhibits hIAPP assembly into amyloid fibrils. (**a**) Unseeded aggregation kinetics of hIAPP (5 μM) in the presence of α-IAPP-O (0.0625, 0.125 and 0.25 μM) and IgG control antibody (0.25 μM). Experimental ThioT fluorescence data are means ± s.e.m. from four replicates. (**b-d**) Kinetic analysis assuming fragmentation-dominated (**b**) or secondary nucleation-dominated (**c**) mechanisms at varying k_+k_-, k_+k_n, and k_+k_2 product rates. Fitting represented by solid lines is consistent with an effect of α-IAPP-O on the product of fibril elongation and primary nucleation rate (k_+k_n) under both fragmentation- and secondary nucleation-dominated regimes (see also Figure 2d), but not on k_+k_- and k_+k_2. Simulated product of fibril elongation and primary nucleation rate for each condition tested (**d**). Shading in grey indicates poor global fit.

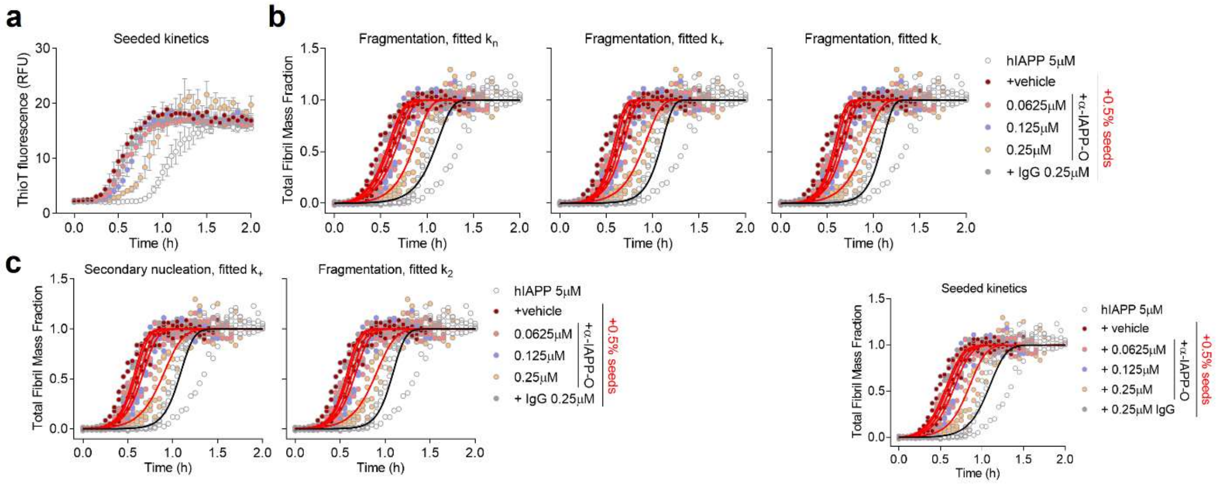

d

		Fragmentation_fitted parameters			Secondary nucleation_fitted parameters		
		Primary nucleation, k_n ($M^{-n_c+1}.min^{-1}$ x10^6)	Elongation, k_+ ($M^{-1}.min^{-1}$ x10^{-3})	Fragmentation, k_- (min^{-1}x10^4)	Primary nucleation, k_n (M^{-n_c+1} $.min^{-1}$x10^6)	Elongation, k_+ ($M^{-1}.min^{-1}$ x10^{-3})	Secondary nucleation, k_2 ($M^{-n_2}.min^{-1}$ x10^5)
hIAPP 5μM (unseeded)		102.712791	153.788793	3.012800	19.127726	26.160689	17.905182
+0.5% seeds	+vehicle	2981.808467	190.605457	3.243208	630.837599	32.082133	19.171988
	+α-IAPP-O 0.0625μM	1812.668214	156.035693	2.653203	376.790881	26.381946	15.757408
	+α-IAPP-O 0.125μM	1264.662782	139.378906	2.368990	255.392313	23.495306	14.028365
	+α-IAPP-O 0.25μM	0.000001	77.174987	1.307727	0.00000007	13.028855	7.759225
	+IgG 0.25μM	2500.025641	176.333405	2.999593	524.665905	29.862633	17.842187

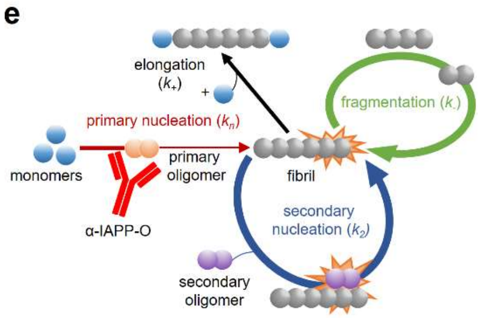

Supplementary Figure 6 α-IAPP-O inhibits primary nucleation. (**a-d**) Seeded hIAPP aggregation kinetics (0.5% fibrillar seeds) in the presence of α-IAPP-O and IgG control antibodies. ThioT fluorescence experimental data (means ± s.e.m. from four replicates) (**a**). Kinetic analysis at varying rates of primary nucleation (k_n), elongation (k_+) and fragmentation (k_-) under fragmentation-dominated (**b**) and secondary nucleation-dominated (**c**) regimes. Fitting to experimental data is represented by solid lines (unseeded and seeded hIAPP aggregation in black and red, respectively). Primary nucleation fitting under secondary nucleation-dominated regime is shown in Figure 2e. Simulated kinetic rates for each condition tested (**d**). (**e**) Schematic illustration of molecular mechanisms involved in hIAPP fibril formation, and inhibition of primary nucleation resulting from the interaction between α-IAPP-O and primary oligomers.

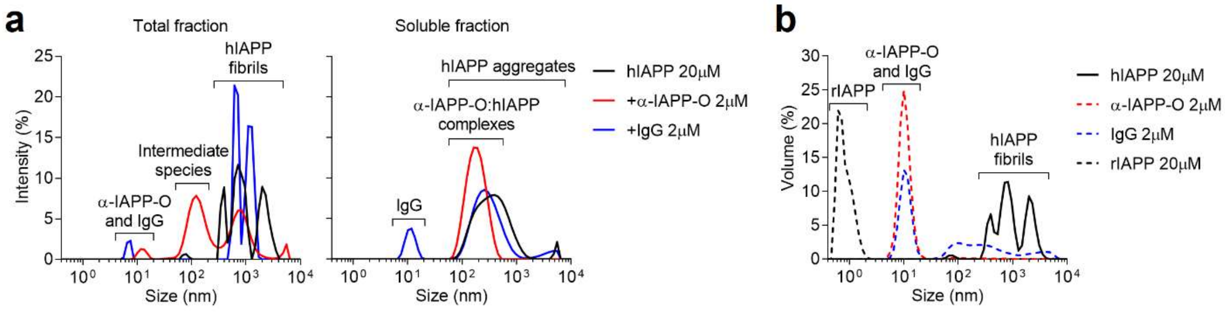

Supplementary Figure 7 α-IAPP-O forms complexes with soluble pre-fibrillar hIAPP species. (**a**) Intensity distribution of particle sizes in total (left panel) and soluble fractions (right panel) using dynamic light scattering (DLS). (**b**) Volume distribution of particle sizes in fractions containing hIAPP fibrils, α-IAPP-O, IgG, and monomeric rodent IAPP (rIAPP) measured by DLS. Data represent the average of three measurements performed on each of the two biological replicates shown in Figure 2h.

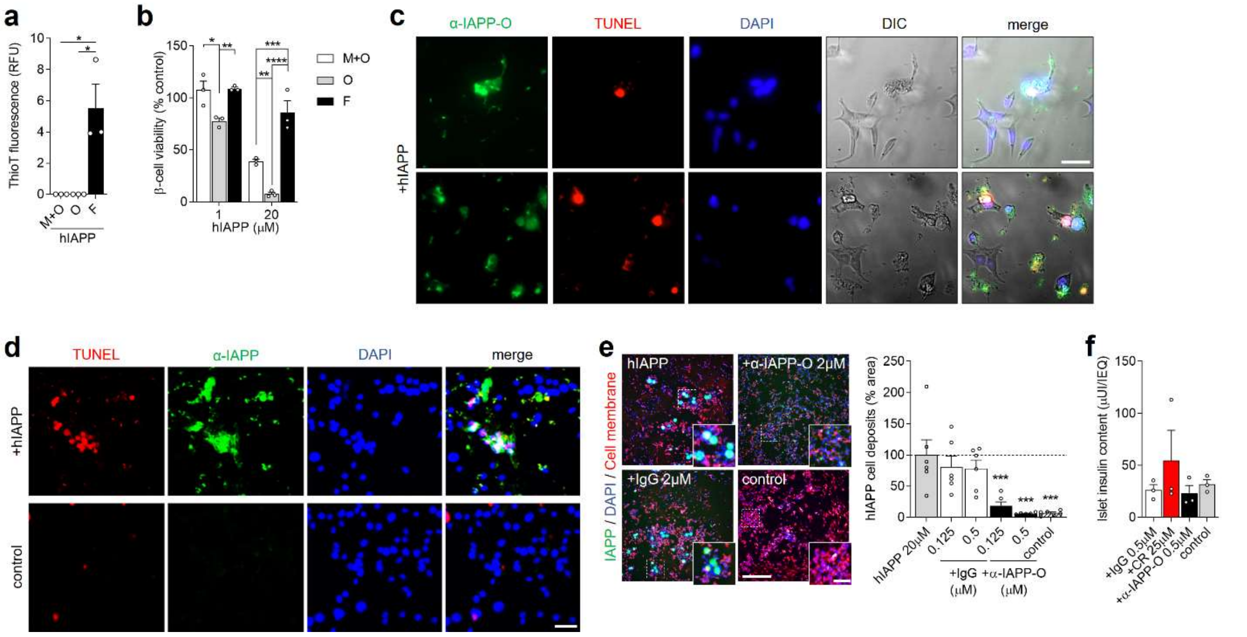

Supplementary Figure 8 α-IAPP-O prevents the deposition of toxic hIAPP oligomers on INS-1 beta cells. (**a**) Amyloid content in samples initially containing 20 μM hIAPP monomers and oligomers (M+O), oligomers (O), and fibril end products (F) using thioflavin-T fluorescence assay. (**b**) Viability of INS-1 beta cells exposed to pre-formed hIAPP species as a function of concentration and assessed by MTT assay. Data in **a**,**b** are from three replicates. (**c**,**d**) Representative fluorescence microscopy images of membrane-bound hIAPP deposits detected by α-IAPP-O (**c**, in green) and a non-selective α-IAPP antibody (**d**, T-4157, in green), and TUNEL-positive apoptotic INS-1 beta cells (in red) upon incubation with hIAPP oligomers (+hIAPP, 20 μM). Cell nuclei are visualized by DAPI (in blue) and cells not exposed to hIAPP oligomers are used as control. DIC, differential interference contrast. Scale bar: 25 μm. (**e**) Representative fluorescence microscopy images (left panel) and quantification (right panel) of hIAPP cell deposits stained with α-IAPP (T-4157, in green) on INS-1 cells exposed to hIAPP oligomers (20 μM) supplemented with α-IAPP-O and IgG control at indicated concentrations. DAPI-positive cell nuclei are shown in blue and plasma membrane in red. Scale bars: 300 and 100 μm. Data in (e) are from two independent experiments performed with three biological replicates (**f**) Human pancreatic islet insulin content upon culture in high glucose (11 mM) for seven days in the presence of α-IAPP-O (+α-IAPP-O 0.5 μM), IgG control (+IgG 0.5 μM) and Congo red (+CR 25 μM). Islets cultured in 5.5 mM glucose for seven days are used as control. Data are from three independent experiments (shown in Figure 3i), each of them using islets from a different donor (detailed in Supplementary Table 2). All data are expressed as means ± s.e.m. Statistical analysis was done using one-way ANOVA followed by Tukey's test (**a, b**) or Dunnett's test (**e, f**). *$p< 0.05$; **$p<0.01$; ***$p<0.001$; ****$p<0.0001$ (**a, b**). ***$p<0.001$ (**e**) (comparing treated groups vs 20 μM hIAPP).

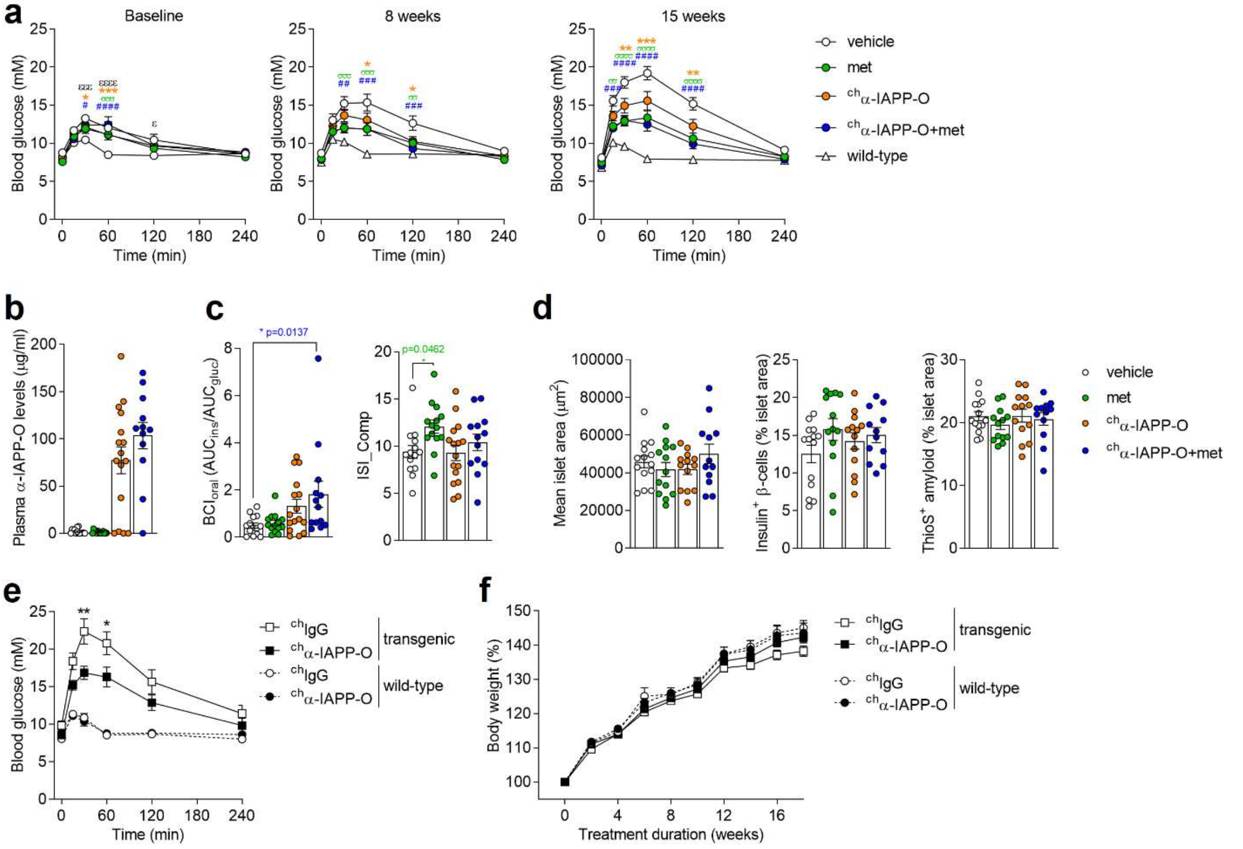

Supplementary Figure 9 Efficacy of α-IAPP-O compared to metformin in transgenic rats. (**a**) Oral glucose tolerance test (oGTT) in naïve transgenic and wild-type rats at baseline (12 weeks of age), latter treated for 8 weeks and 15 weeks with vehicle, rat chimeric α-IAPP-O (^{ch}α-IAPP-O, 3 mg/kg), metformin alone (met; 200 mg/kg/day) or in combination with 3 mg/kg ^{ch}α-IAPP-O (^{ch}α-IAPP-O+met). (**b**) Plasma ^{ch}α-IAPP-O drug levels and (**c**) beta cell function and insulin sensitivity estimates using BCI_{oral} and ISI_Comp methods in 36-week-old hIAPP transgenic rats after 24 weeks of treatment. (**d**) Quantification of islet area, insulin-positive beta cell area and islet amyloid deposition in relation to islet area. Final group numbers in **a-d** were n=14 vehicle, n=14 met (n=13 in d), n=16 ^{ch}α-IAPP-O (n=13 in d), n=13 ^{ch}α-IAPP-O+met (n=12 in d), n=13 wild-type. (**e**) oGTT and (**f**) incremental body weight in transgenic and wild-type rats weekly injected with ^{ch}IgG control antibody (n=12 and n=9, respectively) and ^{ch}α-IAPP-O (n=11 and n=8, respectively) at 3 mg/kg i.p. for 18 weeks. All data are means ± s.e.m.

*p, $^{\#}$p or $^{\varepsilon}$ p<0.05; **p, $^{\#\#}$p or $^{\sigma\sigma}$ p<0.01; ***p, $^{\sigma\sigma\sigma}$p, or $^{\#\#\#}$ p<0.001; $^{\#\#\#\#}$p or $^{\sigma\sigma\sigma\sigma}$ p<0.0001 for (**a**) by repeated measures ANOVA with Dunnett's test (comparing transgenic groups versus wildtype at baseline, and transgenic groups vs vehicle at 8 and 15 weeks), or *p<0.05; **p<0.01 for (**e**) by repeated measures ANOVA with Sidak's test (comparing ^{ch}α-IAPP-O versus ^{ch}IgG). For (**c**) $BCI_{oral} = AUC_{insulin}/AUC_{glucose}$, where AUC corresponds to the area under the glucose ans insulin concentration curves during oGTT. ISI_*Comp* = 10'000 / (√ [G_0 x I_0 x G_{mean} x I_{mean}]), where G_0 and I_0 correspond to fasting blood glucose and plasma insulin levels, and the average levels of glucose (G_{mean}) and insulin (I_{mean}) during oGTT, and p values derived from multiple group comparison vs vehicle after one-way ANOVA and post hoc Dunnett's test.

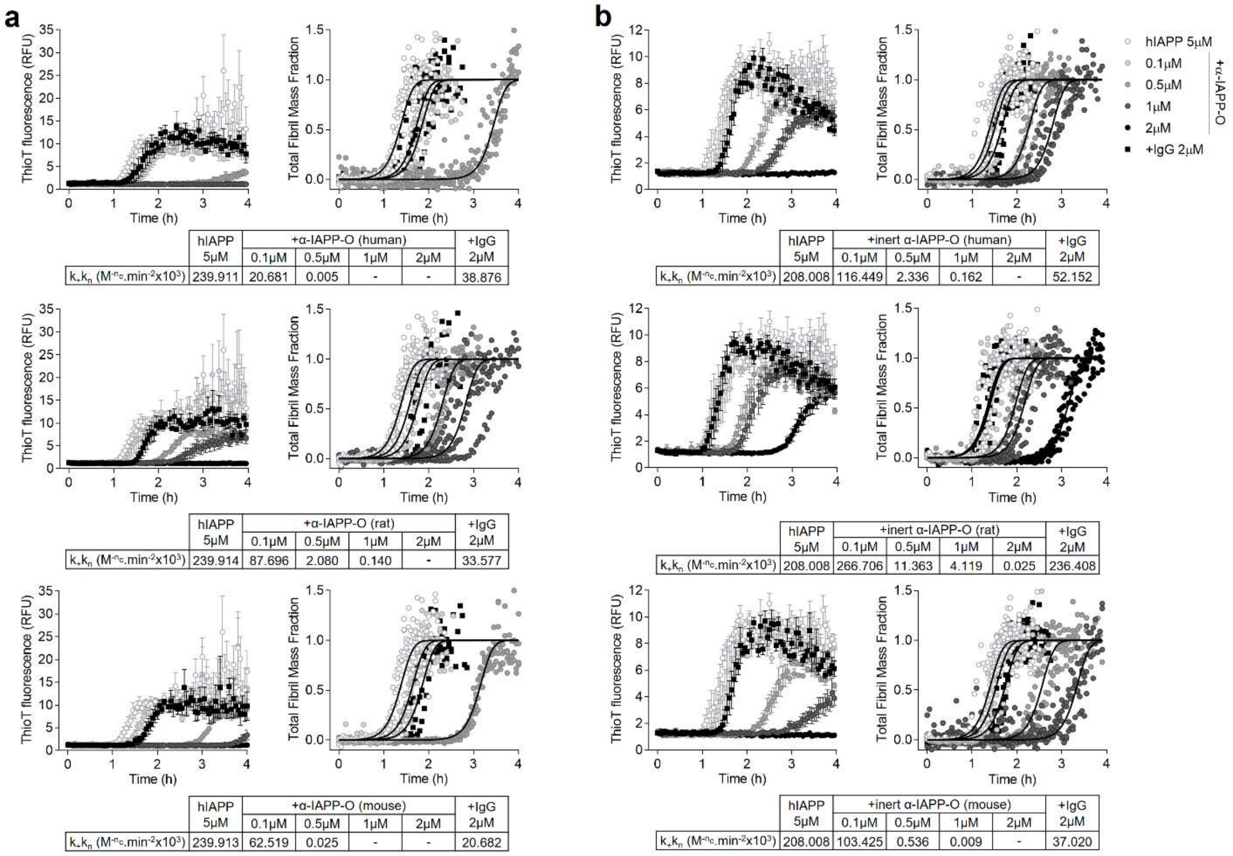

	hIAPP 5μM	+α-IAPP-O (human) 0.1μM	0.5μM	1μM	2μM	+IgG 2μM
k_+k_n ($M^{-n_c}.min^{-2}x10^3$)	239.911	20.681	0.005	-	-	38.876

	hIAPP 5μM	+α-IAPP-O (rat) 0.1μM	0.5μM	1μM	2μM	+IgG 2μM
k_+k_n ($M^{-n_c}.min^{-2}x10^3$)	239.914	87.696	2.080	0.140	-	33.577

	hIAPP 5μM	+α-IAPP-O (mouse) 0.1μM	0.5μM	1μM	2μM	+IgG 2μM
k_+k_n ($M^{-n_c}.min^{-2}x10^3$)	239.913	62.519	0.025	-	-	20.682

	hIAPP 5μM	+inert α-IAPP-O (human) 0.1μM	0.5μM	1μM	2μM	+IgG 2μM
k_+k_n ($M^{-n_c}.min^{-2}x10^3$)	208.008	116.449	2.336	0.162	-	52.152

	hIAPP 5μM	+inert α-IAPP-O (rat) 0.1μM	0.5μM	1μM	2μM	+IgG 2μM
k_+k_n ($M^{-n_c}.min^{-2}x10^3$)	208.008	266.706	11.363	4.119	0.025	236.408

	hIAPP 5μM	+inert α-IAPP-O (mouse) 0.1μM	0.5μM	1μM	2μM	+IgG 2μM
k_+k_n ($M^{-n_c}.min^{-2}x10^3$)	208.008	103.425	0.536	0.009	-	37.020

Supplementary Figure 10 α-IAPP-O variants inhibit hIAPP amyloid fibril formation. (**a**) Aggregation kinetics of hIAPP (5 μM) in the presence of human α-IAPP-O (top), rat chimeric α-IAPP-O (middle) and mouse chimeric α-IAPP-O (bottom). (**b**) Aggregation kinetics of hIAPP (5 μM) in the presence of inert versions of human α-IAPP-O (top), rat chimeric α-IAPP-O (middle) and mouse chimeric α-IAPP-O (bottom). Antibodies were tested at 0.1, 0.5, 1 and 2 μM and an isotype-matched IgG antibody (2 μM) served as internal control in each experiment. Experimental ThioT fluorescence data (left panels in a and b) are means ± s.e.m. from six (hIAPP 5 μM) and three to four replicates (hIAPP + antibodies at various concentrations). Kinetic analysis assuming secondary nucleation-dominated mechanisms with fitting represented by solid lines (right panels in a and b) and product of fibril elongation and primary nucleation rate (k_+k_n) for each condition tested (tables in a and b). -, full inhibition of amyloid fibril formation.

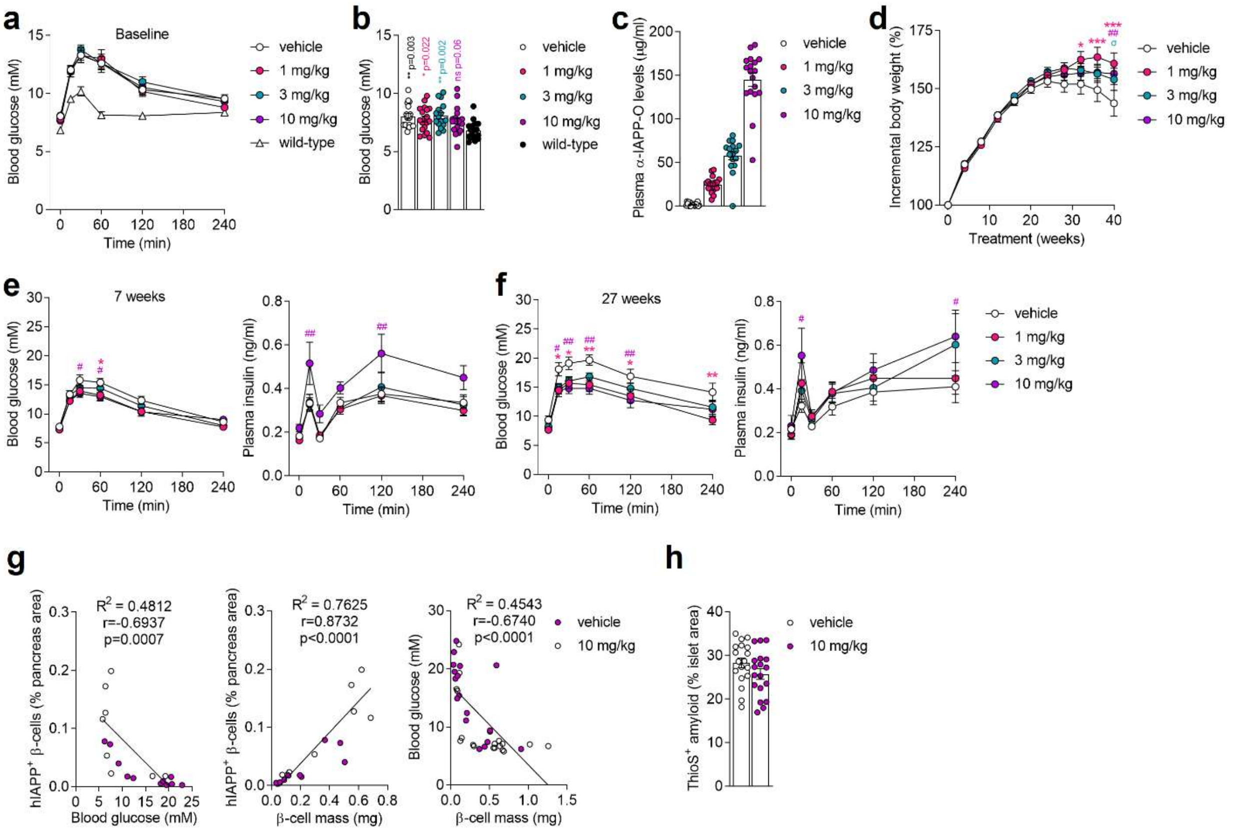

Supplementary Figure 11 Weekly α-IAPP-O dosing in diabetic rats slowed disease progression. (**a**) Oral glucose tolerance test (oGTT) and (**b**) fasting blood glucose levels in naïve transgenic and wild-type at randomization into indicated treatment groups (baseline). (**c**) Averaged ^{ch}α-IAPP-O plasma levels and (**d**) incremental body weight curves in hIAPP transgenic rats upon weekly intraperitoneal (i.p.) injection of rat chimeric α-IAPP-O (^{ch}α-IAPP-O) at 1 mg/kg, 3 mg/kg and 10 mg/kg, and vehicle for 40 weeks. Incremental body weight (%) was expressed in relation to initial body weight measured at 12 weeks of age before the start of the treatment. (**e**) Blood glucose levels and plasma insulin levels during oGTT after 7 weeks of treatment. (**f**) Blood glucose levels and plasma insulin levels during oGTT after 27 weeks of treatment of treatment (supplemental to Figure 4d,e). (**g**) Correlation analyses between pancreatic hIAPP-immunoreactive beta cell content (see Figure 5j,k), fasting blood glucose levels, and pancreatic beta cell mass, and (**h**) quantitative analysis of thioflavin S-positive islet amyloid deposits in transgenic rats weekly injected with vehicle and ^{ch}α-IAPP-O (10 mg/kg i.p.) for 41 weeks Final group numbers in **a**-**h** were n=18 vehicle (n=17 for plasma insulin in **f**, n=11 for hIAPP-positive beta cells vs. blood glucose and beta cell mass in **g**), n=19 1 mg/kg, n=16 3 mg/kg, n=18 10 mg/kg (n=11 for hIAPP-positive beta cells vs. blood glucose and beta cell mass in **g**), n=17 wild-type. All data are means ± s.e.m. Statistical analysis was done using one way ANOVA followed by Dunnett's test for (**b**) with p values indicated in graph. *p, $^{\#}$p or $^{\sigma}$p <0.05; **p or $^{\#\#}$ p<0.01; *** p<0.0001 for **d-f** by repeated measures ANOVA with Dunnett's test (comparing treated groups versus vehicle). For correlation analysis in **g** Pearson correlation coefficient r was calculated, and corresponding p values are reported. For **h** two-tailed unpaired t test was done (t=1.503, df=34, p=0.1420).

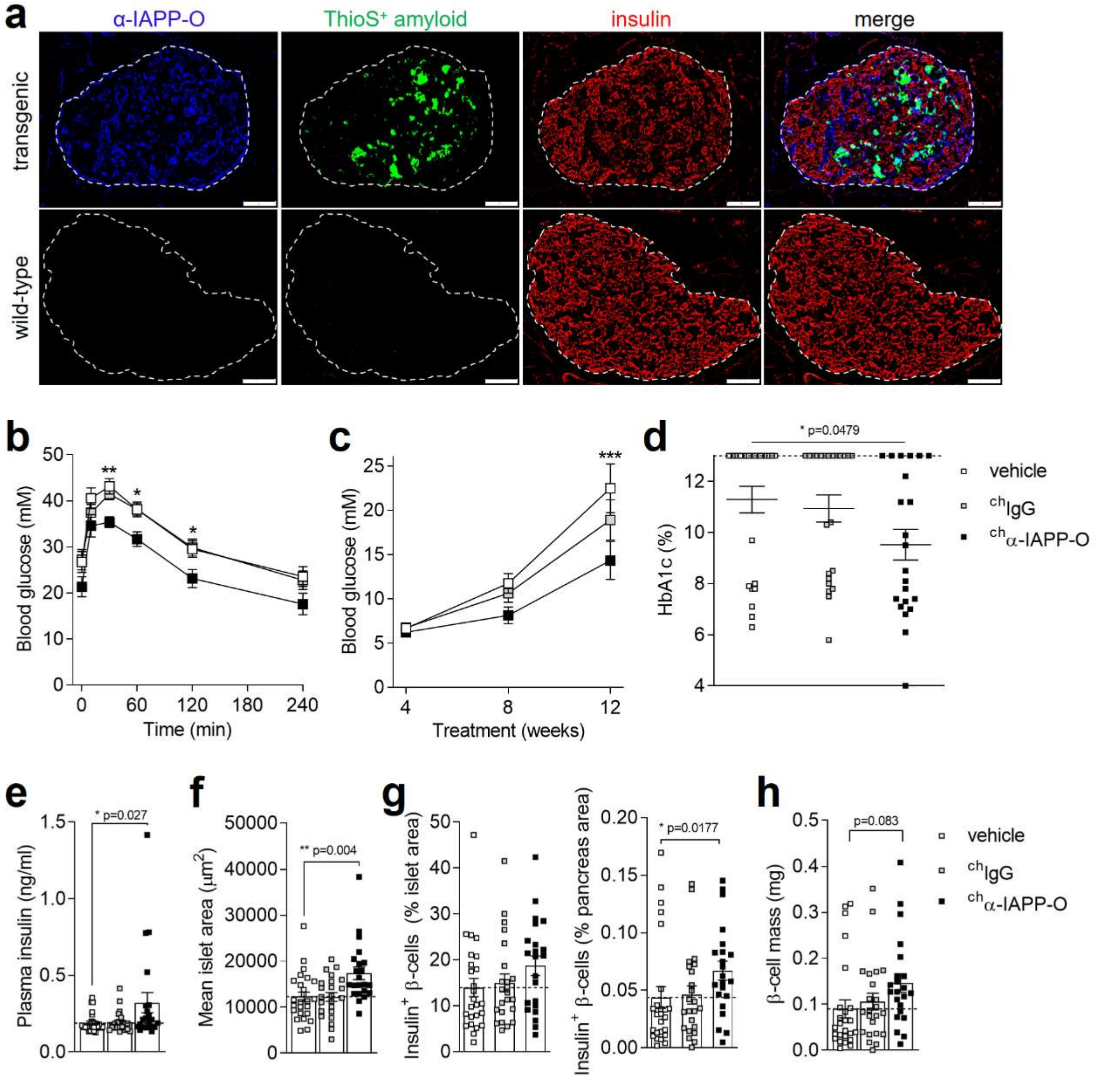

Supplementary Figure 12 α-IAPP-O improves glycemia and protects pancreatic beta cells in hIAPP transgenic mice. (**a**) Representative images of individual pancreatic islets from a transgenic and a wild-type mouse stained for α-IAPP-O target engagement (blue) three day after a single intraperitoneal administration (30 mg/kg). ThioS-positive islet amyloid fibrils (green) and insulin-positive beta cells (red) are shown. Scale bar: 50 µm. (**b**) Blood glucose concentration over time after oral glucose challenge (oGTT) in hIAPP transgenic mice weekly injected (i.p.) with vehicle (n=25), mouse chimeric α-IAPP-O (^{ch}α-IAPP-O, 10 mg/kg, n=22) or an isotype-matched ^{ch}IgG antibody (10 mg/kg, n=23) for 10 weeks. (**c**) Fasting blood glucose levels over the 12-week treatment period in transgenic mice described in **b**, (**d**) glycated haemoglobin HbA1c, and (**e**) fasting plasma insulin levels after 12 weeks of chronic treatment in transgenic mice described in **b**,**c**. Dashed line in **d** represents the upper detection limit of the HbA1c assay. (**f**) Histological analysis of islet size, (**g**) insulin-immunoreactive beta cell area and (**h**) beta cell mass in the pancreas of transgenic mice described in **b-e**. All data are means ± s.e.m. Statistical analysis was done using repeated measures ANOVA followed by Dunnett's test (**b,c**) or one-way ANOVA followed by Dunnett's test (**d-h**) p values indicated in graph. *$p < 0.05$; $^{**}p<0.01$; *** $p<0.0001$ (**b,c**) comparing ^{ch}α-IAPP-O versus vehicle.

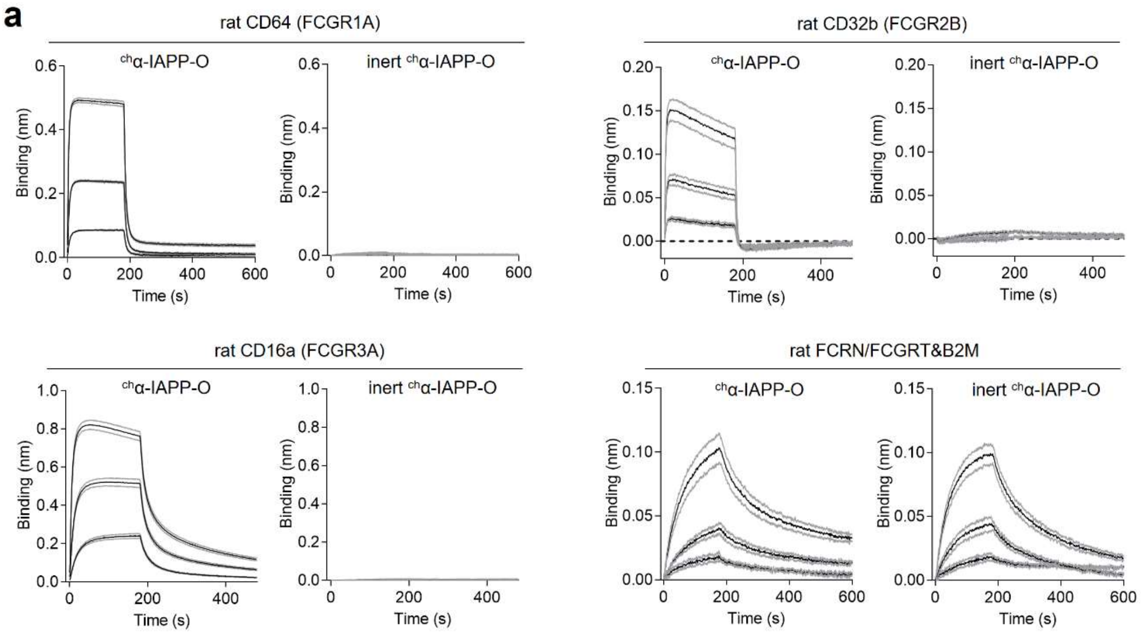

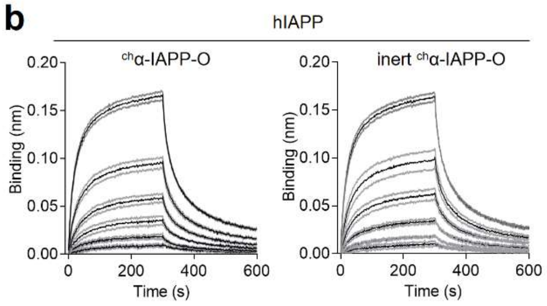

c

K_D (M)	Rat CD64 ($\times10^{-7}$)	Rat CD32b ($\times10^{-7}$)	Rat CD16a ($\times10^{-7}$)	Rat FCRN/ FCGRT &B2M ($\times10^{-7}$)	hIAPP ($\times10^{-9}$)
chα-IAPP-O	3.54 ± 0.33	8.48 ± 0.22	0.36 ± 0.01	0.82 ± 0.04	29.95 ± 0.53
inert chα-IAPP-O	ND	ND	ND	1.32 ± 0.10	32.00 ± 4.36

Supplementary Figure 13 Effector and inert α-IAPP-O interaction to IgG-Fc binding receptors. (**a**) Biolayer interferometry sensorgrams showing interactions between the Fc region of rat chα-IAPP-O and its inert variant with rat IgG-Fc-binding receptors responsible for effector function such as CD64 (FcγRI), CD32b (FcγRIIB) and CD16a (FcγRIIIA) receptors, and the neonatal FcRn involved in antibody recycling. His-tagged recombinant Fc receptors were immobilized on the surface of anti-His biosensors and exposed to rat chα-IAPP-O and inert chα-IAPP-O (666, 222 and 74 nM). (**b**) Binding of rat chα-IAPP-O and inert chα-IAPP-O (100, 50, 25, 12.5, 6.25 and 3.125 nM) to hIAPP immobilized on AR2G biosensors. (**c**) Antibody K_D values for Fc receptors and hIAPP. Data are means ± s.e.m. of quadruplicates. ND, not determined due to low binding signal.

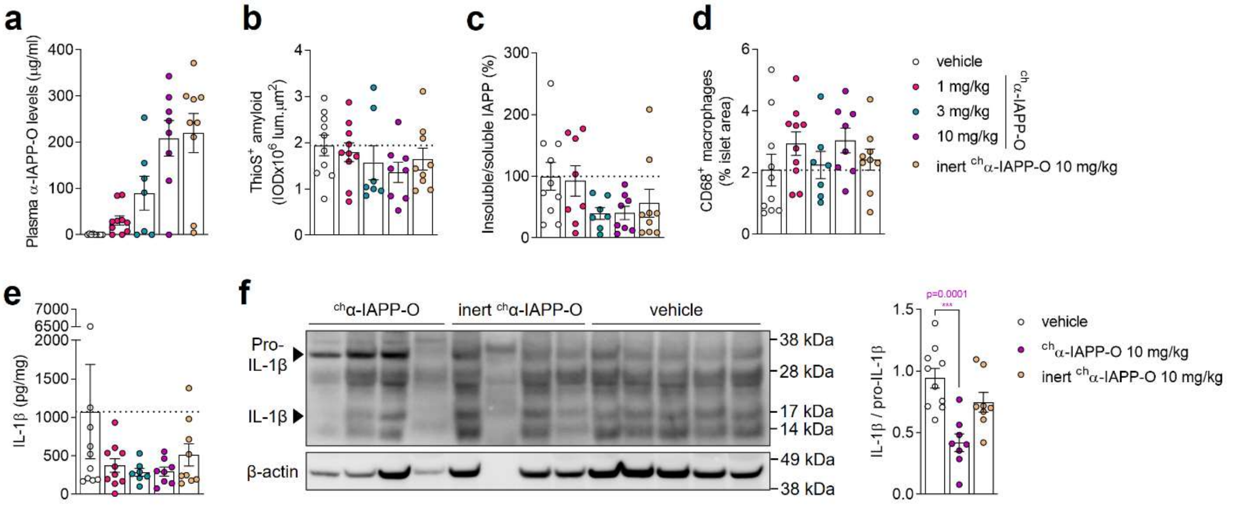

Supplementary Figure 14 Mechanism of action of α-IAPP-O in transgenic rats. (**a**) ^{ch}α-IAPP-O levels in plasma of transgenic rats weekly injected (i.p.) with ^{ch}α-IAPP-O (1 mg/kg, n=10; 3 mg/kg, n=7; 10 mg/kg, n=8), inert ^{ch}α-IAPP-O (10 mg/kg, n=9) and vehicle (n=10) for 38 weeks (supplemental to Figure 5a-d). (**b**) Quantification of ThioS-positive amyloid fibril density (IOD, integrated optical density) within pancreatic islets. (**c**) Insoluble over soluble IAPP content in rat pancreas homogenates measured by ELISA. (**d**) Histological analysis of CD68-immunoreactive islet macrophages. (**e**,**f**) IL-1β cytokine levels in pancreas homogenates of transgenic rats described in **a**-**d** and determined by (**e**) ELISA and (**f**) Western blot. All data are means ± s.e.m.*** p=0.001 by one way ANOVA followed by Dunnett's test (**f**).

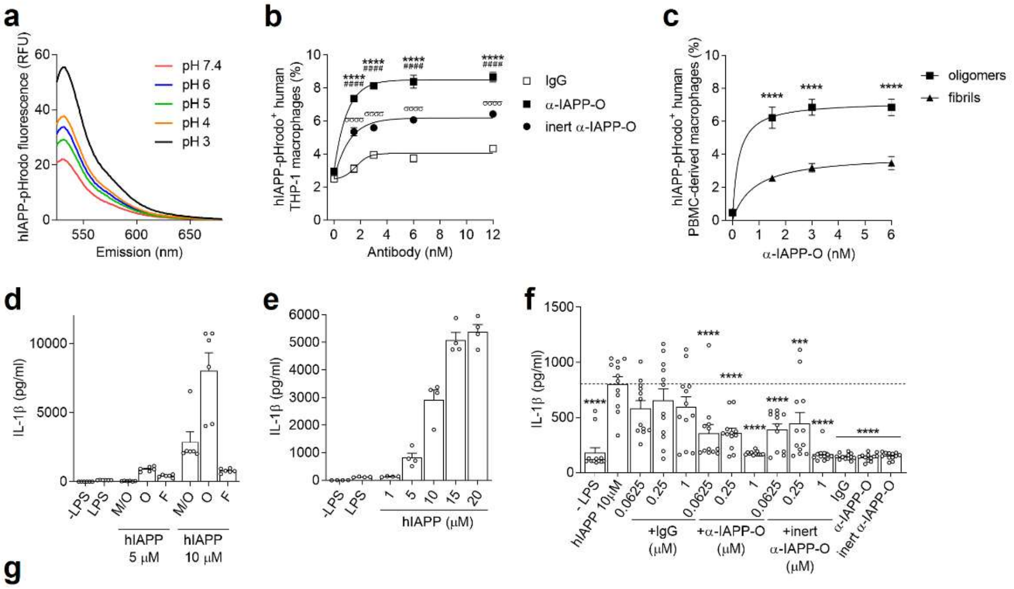

g

K_D (M)	Human CD16a [FCGR3A] (x10^{-8})	Human CD16b [FCGR3B] (x10^{-8})	Human CD32a [FCGR2A] (x10^{-8})	Human CD32b [FCGR2B] (x10^{-8})	Human CD64 [FCGR1A] (x10^{-8})	Human FCGRT &B2M [FCRN] (x10^{-8})	hIAPP (x10^{-9})
α-IAPP-O	7.10 ± 0.07	2.30 ± 0.18	1.27 ± 0.05	1.50 ± 0.10	1.33 ± 0.03	1.56 ± 0.07	14.30 ± 1.34
inert α-IAPP-O	ND	ND	ND	ND	ND	1.91 ± 0.04	15.00 ± 1.26

Supplementary Figure 15 α-IAPP-O triggers the uptake of hIAPP oligomers rather than fibrils by human macrophages and reduces hIAPP oligomer-mediated inflammasome dependent secretion of IL-1β. (**a**) pH-dependent fluorescence of prefibrillar hIAPP aggregates labeled with pHrodo dye (hIAPP-pHrodo, 1 μM). Data are means of two replicates. (**b**) Phagocytosis of hIAPP-pHrodo by human THP-1 macrophages as a function of α-IAPP-O, inert α-IAPP-O and IgG control antibody concentrations using flow cytometry. (**c**) Human PBMC-derived macrophage phagocytic activity on pHrodo-labeled prefibrillar oligomers and amyloid fibrils at increasing α-IAPP-O concentrations. Data in **b**,**c** are means ± s.e.m. of three biological replicates. (**d**-**f**) Human THP-1 macrophage release of pro-inflammatory IL-1β cytokine when exposed to hIAPP. (**d**) IL-1β release in response to 5 and 10 μM pre-formed hIAPP species. Monomers and oligomers, M/O; oligomers (O); amyloid fibrils (F). Data are from two independent experiments performed with three biological replicates. (**e**) IL-1β response as a function of exposure to hIAPP oligomers. Data are from four biological replicates. (**f**) Release of IL-1β from THP-1 macrophages exposed to hIAPP oligomers (hIAPP 10 μM) supplemented with α-IAPP-O, inert α-IAPP-O and IgG control at indicated concentrations. Macrophages were primed with LPS unless stated differently (-LPS). α-IAPP-O and IgG were used at 1 μM in absence of hIAPP. Data are from two independent experiments performed with six biological replicates. (**g**) α-IAPP-O and inert α-IAPP-O K_D values for human Fc receptors and hIAPP determined by biolayer interferometry. His-tagged recombinant Fc receptors (Sino Biological) were immobilized on the surface of anti-His biosensors and hIAPP on AR2G biosensors. Data are from four experiments. ND, not determined due to absent binding signal. All data are means ± s.e.m. ****p, $^{\#\#\#\#}$p or $^{\sigma\sigma\sigma\sigma}$p <0.0001 (**b**) by repeated measures ANOVA with Tukey's test (****comparing test α-IAPP-O vs IgG, $^{\#\#\#\#}$ comparing inert α-IAPP-O vs IgG and $^{\sigma\sigma\sigma\sigma}$ comparing α-IAPP-O vs inert α-IAPP-O). ****p <0.0001 (**c**) by repeated measures ANOVA with Sidaks's test (comparing oligomers versus fibrils). ***p <0.001 and ****p <0.0001 (**f**) by one-way ANOVA with Dunnett's test (comparing treatment groups vs hIAPP 10 μM).

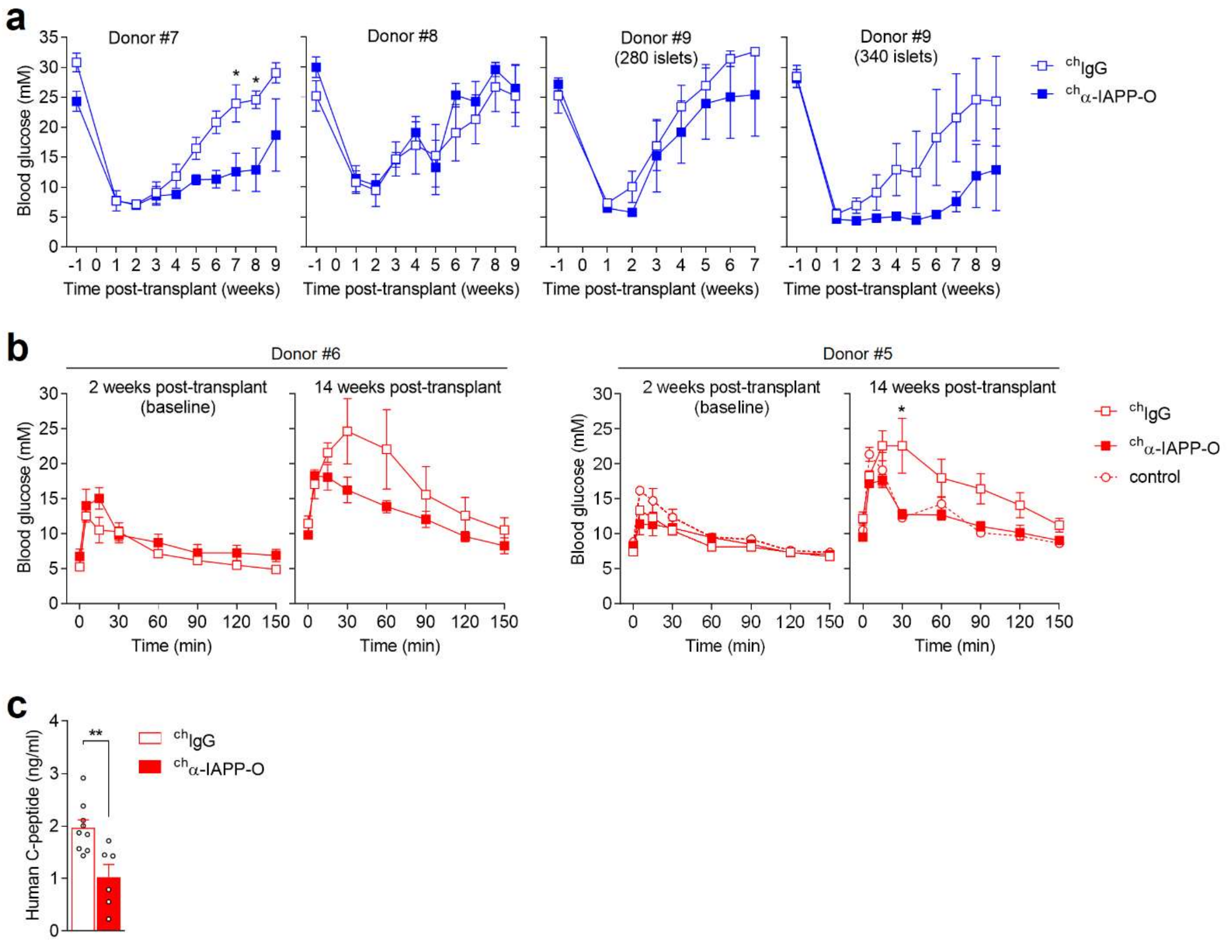

Supplementary Figure 16 α-IAPP-O treatment in human islet-engrafted mice. (**a**) Recurrence of hyperglycaemia in NSG recipient mice weekly injected (10 mg/kg i.p.) with mouse chimeric ^{ch}α-IAPP-O and ^{ch}IgG control (supplemental to Figure 6b). Data are from three independent experiments using human islets preparations from three non-diabetic donors (see Supplementary Table 2): donor #7 (n=2 ^{ch}IgG and n=3 ^{ch}α-IAPP-O), donor #8 (n=4 ^{ch}IgG and n=3 ^{ch}α-IAPP-O), donor #9 (280 and 340 islets, n=4 and 3 ^{ch}IgG, n=4 and 4 ^{ch}α-IAPP-O). Blood glucose values represent the average of biweekly measurements. (**b**) Blood glucose concentration during oral glucose tolerance tests 2 weeks (baseline) and 14 weeks post-transplant in HFD-fed Rag2$^{-/-}$ recipient mice weekly injected (10 mg/kg i.p.) with ^{ch}α-IAPP-O and ^{ch}IgG control for 12 weeks (2 to 14 weeks post-transplant; supplemental to Figure 6d). Naïve Rag2$^{-/-}$ recipient mice kept on regular diet for 12 weeks served as controls. Data are from two independent experiments using human islets preparations from two pre-diabetic donors (see Supplementary Table 2): donor #6 (n=3 ^{ch}IgG and n=3 ^{ch}α-IAPP-O) and donor #5 (n=6 ^{ch}IgG, n=3 ^{ch}α-IAPP-O and n=2 control). (**c**) Human C-peptide plasma levels in HFD-fed Rag2$^{-/-}$ recipient mice weekly injected with ^{ch}α-IAPP-O (10 mg/kg i.p., n=6) and ^{ch}IgG control (10 mg/kg i.p., n=9) for 12 weeks. All data are means ± s.e.m. * $p<0.05$ (**a,b**) by repeated measures ANOVA with Sidak's comparing ^{ch}α-IAPP-O vs ^{ch}IgG, and (**c**) two-tailed unpaired t test with **$p<0.05$ comparing ^{ch}α-IAPP-O vs ^{ch}IgG.

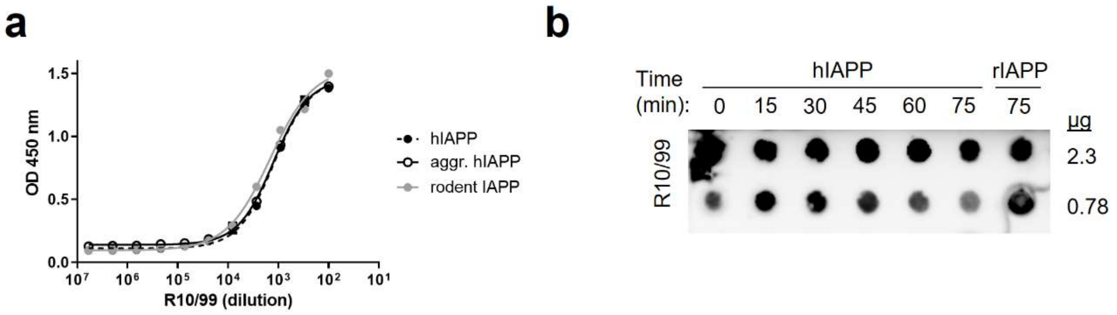

Supplementary Figure 17 (**a**) Direct ELISA with mouse monoclonal antibody R10/99 versus freshly coated hIAPP (hIAPP), pre-aggregated hIAPP (aggr. hIAPP) and rodent IAPP (rIAPP). Lines indicate sigmodal curve fits of R10/99 dilution series vs absorbance. (**b**) Dot blot analysis of hIAPP and rIAPP fractions collected over the course of amyloid formation as described in Figure 2 and detected with non-selective α-IAPP antibody R10/99.

Supplementary Tables

Analyte	Antigen	K_D (nM)	k_a ($M^{-1}s^{-1}x10^5$)	k_d ($s^{-1}x10^{-4}$)	R^2
α-IAPP-O	hIAPP	1.413	7.243	10.230	0.994
		1.563	5.699	8.910	0.992
		1.661	5.533	9.189	0.996
		1.546 ± 0.072	6.158 ± 0.544	9.443 ± 0.401	
	biotin-hIAPP	2456	0.00362	8.905	0.996
		2698	0.00329	8.880	0.997
		3374	0.00211	7.142	0.997
		2843 ± 274	0.0030 ± 0.0004	8.309 ± 0.583	
α-IAPP-O Fab	hIAPP	5686	0.190	1085	0.993
		7067	0.170	1207	0.995
		6673	0.179	1200	0.995
		6475 ± 410	0.179 ± 0.005	1164 ± 39	

Supplementary Table 1 Biolayer interferometry binding constants of α-IAPP-O antibody and α-IAPP-O-derived Fab fragment to surface-immobilized hIAPP aggregates and monomeric biotin-hIAPP. K_D, affinity constant; k_a, association constant; k_d, dissociation constant; R^2, goodness of fitting using 1:1 interaction model. Data are expressed as means ± s.e.m. from three independent runs.

Donor demographics										
Donor		#1	#2	#3	#4	#5	#6	#7	#8	#9
Mean age in years (range)		55.33 (39 – 72)								
Sex (M/F)		F	F	M	M	M	M	F	F	F
Mean BMI in kg/m^2 (range)		30.01 (21.0 – 40.6)								
Mean %HbA$_{1c}$ (range)		5.8 (5.3 – 6.4)								
Cause of death		Stroke						NDD-Neurological	CVA	CVA
Diabetes? (Y/N)		N						N		
Pancreas										
Cold ischaemia time in hours (range)		8.9 (3.9 – 21.5)								
Islet handling and use										
Estimated purity (%)		80	90	-	80	70	60	95	90	90
Experimental islet use (including in which experiment each islet preparation was used)	Culture + insulin secretion	X	X	X						
	Culture + histology	X	X		X					
	Transplant into Rag2$^{-/-}$ mice (age in weeks; islets/mouse; treatment start in weeks post-transplant)		57; 450; 8			18; 450; 2	13; 450; 2			
	Transplant into NSG mice (age in weeks; islets/mouse)							12; 330	20; 320	6-8; 280 and 340
Experimental site		University of Lille (France)						University of British Columbia (Vancouver, Canada)		

Supplementary Table 2 Human islet donor characteristics. BMI≥30 kg/m^2 indicate obesity and HbA1c levels between 5.7 and 6.4 % a higher risk of developing type 2 diabetes. This table was prepared following recommendations by Hart and Powers[2]. NDD: neurological determination of death, CVA: cerebrovascular accident.

Analyte	Antigen	K_D (nM)	k_a ($M^{-1}s^{-1}x10^5$)	k_d ($s^{-1}x10^{-4}$)	R^2
α-IAPP-O (human)	hIAPP	21.86	2.843	62.14	0.982
		23.56	2.679	63.13	0.987
		23.65	2.623	62.03	0.983
		23.02 ± 0.58	2.715 ± 0.066	62.43 ± 0.35	
^{ch}α-IAPP-O (rat)		27.10	2.791	75.64	0.984
		29.44	2.784	81.97	0.986
		28.05	2.832	79.44	0.983
		28.20 ± 0.67	2.802 ± 0.014	79.02 ± 1.84	
^{ch}α-IAPP-O (mouse)		17.00	2.447	41.60	0.985
		17.90	2.601	46.54	0.986
		17.02	2.562	43.62	0.981
		17.31 ± 0.29	2.537 ± 0.046	43.92 ± 1.43	

Supplementary Table 3 Biolayer interferometry binding constants of human α-IAPP-O, rat and mouse chimeric α-IAPP-O (^{ch}α-IAPP-O) antibodies to hIAPP aggregates immobilized on AR2G sensors. K_D, affinity constant; k_a, association constant; k_d, dissociation constant; R^2, goodness of fitting using 1:1 interaction model. Data are expressed as means ± s.e.m. from three independent runs.

Supplementary Methods

Immunohistochemistry on human pancreas

Paraffin-embedded human pancreas sections (5 µm; obtained from University Hospital Basel, Switzerland) were deparaffinized, rehydrated, and immersed in antigen retrieval solution (70% formic acid) for 10 min. An indirect IHC technique using an automated staining method was used for pancreas sections stained with FITC-conjugated human α-IAPP-O antibody to avoid detection of endogenous human antibodies. Briefly, sections were blocked in Protein Block solution (Biosystems) for 10 min followed by PBS + 5% normal goat serum + 1% BSA for 10 min, and incubated for 1h at room temperature with 0.1 and 1 µg/ml FITC-conjugated human α-IAPP-O and FITC-conjugated human isotype control (IgG) antibody labeled using the Pierce FITC Antibody Labeling Kit (Thermo Scientific). Peroxidase-based staining was performed using an HRP-conjugated rabbit anti- FITC antibody (1:30'000 for 30 min; OriGene) combined with Bond Polymer Refine Detection Kit (Biosystems). Slides were mounted using Pertex (Biosystems) and bright-field imaging was performed on a Dotslide VS120 slide scanner (Olympus).

Determination of α-IAPP-O cross-reactivity by ELISA

96-well microplates (Corning) were coated with freshly reconstituted synthetic hIAPP and amyloidogenic peptides resuspended at 1-10 µg/ml in carbonate buffer (15 mM Na_2CO_3, 35 mM $NaHCO_3$, pH 9.4) or PBS overnight at 4°C. Unspecific binding sites were blocked with PBS + 0.1% Tween-20 + 2% BSA and recombinant human α-IAPP-O antibody (20, 4 and 0.8 nM) was applied for 1h at room temperature. Binding was determined using a donkey anti-human HRP-conjugated secondary antibody (#709-036-098, 1:2000; Jackson ImmunoResearch) followed by measurement of HRP activity at 450 nm (optical density, OD).

Immunohistochemistry on human brain

Paraffin-embedded human brain sections from donors with Alzheimer's disease (AD) were deparaffinized, rehydrated, blocked in PBS + 5% serum (horse/goat) + 4% BSA for 1h, incubated with mouse chimeric ^{ch}α-IAPP-O (100 nM) and mouse anti-Aβ-amyloid 6E10 antibody (30 nM; BioLegend) overnight at 4°C, and stained with biotinylated donkey anti-mouse secondary antibody (#715-065-150, 1:500; Jackson ImmunoResearch) combined with signal amplification using Vectastain ABC kit (Vector Laboratories). Slides were mounted using Hydromount media (National Diagnostics) and imaged on a Dotslide VS120 slide scanner (Olympus).

Epitope mapping

Precision epitope mapping was conducted at Pepscan Presto BV (Zuidersluisweg 2, 8243RC Lelystad, The Netherlands) using Pepscan's proprietary CLIPS technology for conformational epitopes[3,4]. The individual contribution of hIAPP amino acid residues 1-22 to α-IAPP-O binding was assessed by full substitution mutagenesis. Sensitivity of α-IAPP-O binding to peptide length and structure was assessed using C-terminus truncated hIAPP peptides (6 to 26 residues) and by addition of 0.1% SDS.

IAPP immunostaining on INS-1 cells

Rat insulinoma INS-1 beta cells (INS-1 832/13, SCC207, Sigma) were exposed to hIAPP aggregates, α-IAPP-O, IgG control or vehicle for 12h and fixed in 4% paraformaldehyde for 10 min at room temperature, washed in PBS, blocked with a solution containing 5% serum (horse/goat) + 4% BSA in PBS for 1h, stained with rabbit anti-IAPP antibody (α-IAPP, 1:1000; T-4157, Peninsula Laboratories) for 2h at room temperature and with Cy2-conjugated donkey

anti-rabbit secondary antibody (#711-225-152, 1:1000; Jackson ImmunoResearch) for 1h at room temperature. Cells were also stained with CellBrite^{TM} Red cytoplasmic membrane dye (5 µl/ml in PBS; #30023, Biotium) for 20 minutes at room temperature and with DAPI (1:1000 in PBS). IAPP aggregates were also stained on living cells exposed to human α-IAPP-O (200 nM) for 1h prior to fixation and detected using Cy2-conjugated donkey anti-human secondary antibody (#709-225-149, 1:250; Jackson ImmunoResearch). Cell apoptosis was visualized by TUNEL staining (In Situ Cell Death Detection Kit, TMR red; Roche). Cells were kept in PBS and fluorescence images were captured using a confocal laser scanning microscope (Leica SP8) and a widefield fluorescence imaging system (IN Cell Analyzer 2500 HS, GE Healthcare). Image analysis was performed on at least 3 different fields of view per well and 3 wells per condition using Image J software. IAPP deposition was computed as the image area occupied by IAPP staining expressed as percentage, with 100% corresponding to cells exposed to 20 µM hIAPP in absence of antibody.

Plasma α-IAPP-O levels

Plasma was isolated from rat blood samples by centrifugation and rat chimeric ^{ch}α-IAPP-O levels were determined by direct ELISA. 96-well microplates (Corning) were coated overnight at room temperature with an anti-idiotypic Fab antibody fragment (1 µg/ml in PBS, custom-made, AbD Serotec) generated by HuCAL technology for high affinity binding to α-IAPP-O. Non-specific binding sites were blocked with PBS + 0.1% Tween-20 + 2% BSA for 1h at room temperature. Plasma samples were diluted 1:5’000 to 1:30’000 in PBS, incubated on plate for 1h at room temperature, and ^{ch}α-IAPP-O was detected with HRP-conjugated mouse anti-rat IgG2b (gamma chain specific, #3070-05) and mouse anti-rat kappa (kappa chain specific, #3090-05) secondary antibodies (1:5000, SouthernBiotech), followed by measurement of HRP activity using standard

procedure. Plasma α-IAPP-O levels were calculated based on a standard curve (^{ch}α-IAPP-O concentration ranging from 1.37 pM to 1 nM in PBS).

Transgenic mice

Hemizygous hIAPP transgenic male mice (FVB/N-Tg(Ins2-IAPP)RHFSoel/J, #008232, The Jackson Laboratory, USA) were bred with DBA/2J wild-type female mice (#000671, The Jackson Laboratory, USA) to generate hIAPP transgenic and wild-type F1 male mice on a mixed FVB/N and DBA/2J background that were housed under controlled conditions (21±1ºC, 12:12 hour light/dark cycle with light phase from 2:00 am to 2:00 pm) with free access to standard chow diet (Extrudate 3436, KLIBA NAFAG, Switzerland) and water. Mice received a once-weekly intraperitoneal (i.p.) injection of recombinant mouse chimeric ^{ch}α-IAPP-O and IgG control antibody (10 mg/kg). Treatment was initiated at 4 weeks of age for a duration of 12 weeks.

Mouse oral glucose tolerance test (oGTT) was performed upon a 5 hour-fast (with free access to water) with oral gavage of 2 g/kg glucose (50% solution, B. Braun Medical AG) and repeated blood sampling (0, 10, 30, 60, 120 and 240 min) from the tail vein. Fasting blood glucose was measured after overnight (12h) fasting. Blood glucose and glycated hemoglobin (HbA1c) were measured using a Contour XT glucometer (Bayer) and A1CNow+ test kit (Bayer), respectively. Plasma was isolated by centrifugation and insulin levels were determined by ELISA (mouse insulin ELISA, Mercodia). Mice were euthanized by ketamine/xylazine injection (100/20 mg/kg i.p.; Graeub AG, Switzerland) and pancreas tissue was removed, weighted, fixed in 4% paraformaldehyde (w/v) and embedded in paraffin, or frozen in OCT medium (following soaking in 30% sucrose) for histological analyses. Pancreas sections (3 μm) were blocked in PBS + 2.5% horse serum + 2.5% goat serum + 4% BSA for 1h at room temperature, incubated with guinea pig

polyclonal anti-insulin antibody (1:3, FLEX, Dako) overnight at 4°C and with TRITC-conjugated goat anti-guinea pig secondary antibody (#106-025-003, 1:200, Jackson ImmunoResearch) for 1h at room temperature. α-IAPP-O-bound hIAPP aggregates (three days after a single i.p. administration of 30 mg/kg human α-IAPP-O) were revealed on fresh frozen tissue sections using Cy5-conjugated donkey anti-human secondary antibody (#709-175-149, 1:200, Jackson ImmunoResearch). Slides were mounted using Hydromount media (National Diagnostics) and imaged on a Leica SP8 confocal laser scanning microscope. Islet area and insulin-immunoreactive beta cell content were quantified from at least 27 identified per section on a total of five sections per mouse. Data were computed as the fluorescence area above a predetermined threshold using Image-Pro Premier software (Media Cybernetics) and expressed as percentage of corresponding islet and pancreas area. Beta cell mass (mg) was calculated as follows: (Σ insulin-positive area / pancreas area) x pancreas weight (mg). Mouse experiments were approved by the Veterinary Office of the Canton Zurich, Switzerland (authorization number 150/2012) and performed as recommended by the Federal Veterinary Office (FVO).

Fc gamma receptor binding analysis

Antibody binding to rat and human Fc gamma receptors (FcγRs; Sino Biological, China) composed of His-tagged FCGR1A/CD64 (#80016-R08H and 10256-H08H), His-tagged FCGR2A/CD32a (#10374-H08H), His-tagged FCGR2B/CD32b (#80018-R08H and 10259-H08H), His-tagged FCGR3A/CD16a (#80017-R08H and 10389-H08H), His-tagged FCGR3B/CD16b (#11046-H08H) and untagged FCGRT&B2M (#CT030-R08H and #CT009-H08H) was measured with an Octet RED96 instrument (Pall ForteBio). FcγRs were reconstituted at 50 μg/mL in PBS (pH 7.4) and loaded on anti-His (HIS2) biosensors (Pall ForteBio) according to manufacturer's recommendations. Untagged FCGRT&B2M were reconstituted at 50 μg/mL in

PBS (pH 6) and loaded on amine-reactive (AR2G) biosensors (Pall ForteBio). Rat chimeric $^{ch}\alpha$-IAPP-O and inert $^{ch}\alpha$-IAPP-O, and human α-IAPP-O and inert α-IAPP-O were tested at indicated concentrations in PBS (pH 7.4 or 6.0) and binding response was analyzed with simultaneous Ka/Kd global fitting (1:1 interaction model) using the Octet system software. BLI sensorgrams were drawn using Prism 9 software from GraphPad (San Diego, USA).

Measurement of IL-1β in rat pancreas

ELISA. Flash frozen rat pancreas samples were homogenized (10%, w/v) in 0.9% NaCl + protease inhibitor cocktail (cOmplete™, Roche) and centrifuged (12'000 g, 4°C, 15 min). Rat IL-1β was quantified in the supernatant by ELISA (#BMS630TEN, ThermoFisher Scientific). *Western blotting*. Flash frozen rat pancreas tissues were homogenized (10%, w/v) in modified RIPA buffer (50 mM Tris-HCl pH 7.4, 150 mM NaCl, 1 mM EDTA, 5% NP-40, 0.5% Sodium deoxycholate, 0.1% SDS) and centrifuged (12'000 g, 4°C, 15 min). Proteins in the supernatant (60 µg) were resolved by gradient SDS-PAGE (NuPAGE 4-12% Bis-Tris gels, ThermoFisher Scientific) using NuPAGE LDS sample buffer (ThermoFisher Scientific) + 2.5% β-mercaptoethanol (95°C, 5 min) and NuPAGE MES SDS running buffer (ThermoFisher Scientific), and electroblotted (iBlot 2 Dry Blotting System, ThermoFisher Scientific) onto PVDF membrane. Non-specific binding sites were blocked with PBS + 0.05% Tween® 20 (P7949, Sigma) + 2% BSA (A8022, Sigma) for 1h. Membrane was immunoblotted with a rabbit anti-IL-1β antibody (1:2'500, ab9722, Abcam) and a mouse anti-β-actin antibody (1:100'000, A1978, Sigma) in blocking buffer at 4°C overnight and for 1h at room temperature, washed in PBS + 0.05% Tween® 20, and incubated for 1h with goat anti-rabbit and anti-mouse IgG (H+L) secondary antibodies coupled to HRP (1:10'000, #111-035-144 and #115-035-146, Jackson ImmunoResearch). Antibody binding was revealed with HRP substrate (Pierce™ ECL,

ThermoFisher Scientific) and imaged using an ImageQuant LAS 4000 system (GE Healthcare, Switzerland). Images were quantified using Image J software.

THP-1 macrophages

Phagocytosis assay. Human THP-1 monocytic cells (Cat# TIB-202, ATCC) were seeded at a density of 5 x 10^5 cells/ml and differentiated in RPMI-1640 medium (30-2001, ATCC) supplemented with 100 µg/ml penicillin/streptomycin, 10% FBS and 25 ng/mL PMA (Sigma) for 72h at 37°C and 5% CO_2. IFN-γ (20 ng/mL) was added to the culture medium for the last 24 hours. Lyophilized pHrodo-labeled hIAPP was prepared as described in online methods and pH-dependent fluorescence was measured on a Varioskan LUX plate reader (ThermoFisher Scientific) with fluorescence excitation at 505 nm and emission recorded at 525 to 680 nm (12 nm bandwidth). pHrodo-labeled hIAPP and antibodies were added to THP-1 macrophages plated in fresh RPMI-1640 medium supplemented with 100 µg/ml penicillin/streptomycin and 50 µg/mL of the scavenger receptor inhibitor Fucoidan (F5631, Sigma) for 1h at 37°C. THP-1 macrophages were detached and phagocytosis was analyzed using a FACS Aria II flow cytometer equipped with BD FACS Diva software (BD Biosciences) as described for PBMC-derived macrophages in online methods.

IL-1β release by human THP-1 cells

Human THP-1 cells were seeded at a density of 1.25 x 106 cells/ml in 48-well culture-treated plates (TPP), differentiated in culture medium (RPMI-1640 + 100 µg/ml penicillin/streptomycin + 10% FBS) supplemented with 150 ng/mL PMA (Sigma) for 2h, and placed back in culture medium at 37°C and 5% CO2 overnight. Lyophilized hIAPP was reconstituted at indicated concentrations in serum-free RPMI 1640 medium (11879-RPMI, ThermoFisher Scientific)

supplemented with 100 µg/ml penicillin and 100 µg/ml streptomycin, and incubated for 0h (M/O), 2h (O) and 4h (F) at room temperature under quiescent conditions before being applied to THP-1 cells primed with 500 ng/mL LPS (Sigma) for 3h. Human α-IAPP-O or IgG control antibody were added at indicated concentrations to the peptide solution. Control cells (with and without priming) were incubated in peptide-free culture medium. After 16h, IL-1β release and cytotoxicity were assessed in the cell supernatant using ELISA (ab108865, Abcam) and LDH assay (CyQUANTTM LDH Cytotoxicity assay kit, ThermoFisher Scientific) according to manufacturer's instructions.

Supplementary References

1. Westermark, P., Andersson, A. & Westermark, G. T. Islet amyloid polypeptide, islet amyloid, and diabetes mellitus. *Physiol. Rev.* **91**, 795–826 (2011).
2. Hart, N. J. & Powers, A. C. Use of human islets to understand islet biology and diabetes: progress, challenges and suggestions. *Diabetologia* **62**, 212–222 (2019).
3. de Weers, M. *et al.* Daratumumab, a novel therapeutic human CD38 monoclonal antibody, induces killing of multiple myeloma and other hematological tumors. *J. Immunol.* **186**, 1840–1848 (2011).
4. Niederfellner, G. *et al.* Epitope characterization and crystal structure of GA101 provide insights into the molecular basis for type I/II distinction of CD20 antibodies. *Blood* **118**, 358–367 (2011).
