# Supplementary material for: A human antibody against pathologic IAPP aggregates protects beta cells in type 2 diabetes models
Source: Nat Commun. 2023 Oct 9;14:6294. doi: 10.1038/s41467-023-41986-0 (PMC10562398; doi:10.1038/s41467-023-41986-0)
Supplement: Supplementary file 3 — Reporting Summary [file 41467_2023_41986_MOESM3_ESM.pdf]

nature portfolio

Corresponding author(s): Jan Grimm

Last updated by author(s): Sep 14, 2023

Reporting Summary

Nature Portfolio wishes to improve the reproducibility of the work that we publish. This form provides structure for consistency and transparency in reporting. For further information on Nature Portfolio policies, see our Editorial Policies and the Editorial Policy Checklist.

Statistics

For all statistical analyses, confirm that the following items are present in the figure legend, table legend, main text, or Methods section.

n/a	Confirmed	
[ ]	[x]	The exact sample size (*n*) for each experimental group/condition, given as a discrete number and unit of measurement
[ ]	[x]	A statement on whether measurements were taken from distinct samples or whether the same sample was measured repeatedly
[ ]	[x]	The statistical test(s) used AND whether they are one- or two-sided<br>*Only common tests should be described solely by name; describe more complex techniques in the Methods section.*
[x]	[ ]	A description of all covariates tested
[ ]	[x]	A description of any assumptions or corrections, such as tests of normality and adjustment for multiple comparisons
[ ]	[x]	A full description of the statistical parameters including central tendency (e.g. means) or other basic estimates (e.g. regression coefficient) AND variation (e.g. standard deviation) or associated estimates of uncertainty (e.g. confidence intervals)
[ ]	[x]	For null hypothesis testing, the test statistic (e.g. *F*, *t*, *r*) with confidence intervals, effect sizes, degrees of freedom and *P* value noted<br>*Give P values as exact values whenever suitable.*
[x]	[ ]	For Bayesian analysis, information on the choice of priors and Markov chain Monte Carlo settings
[x]	[ ]	For hierarchical and complex designs, identification of the appropriate level for tests and full reporting of outcomes
[x]	[ ]	Estimates of effect sizes (e.g. Cohen's *d*, Pearson's *r*), indicating how they were calculated

Our web collection on statistics for biologists contains articles on many of the points above.

Software and code

Policy information about availability of computer code

Data collection	Dotslide VS120 slide scanner (Olympus); SP8 confocal laser scanning microscope (Leica); Octet RED96 BLI system (Pall ForteBio); Varioskan LUX plate reader (ThermoFisher Scientific); CM100 transmission electron microscope (Philips); ImageQuant LAS 4000 (GE Healthcare); Zetasizer Nano DLS system (Malvern); IN Cell Analyzer 2500 HS (GE Healthcare); Access Immunoassay system (Beckman Coulter); Contour XT glucometer (Bayer); A1CNow+ test kit (Bayer); FACS Aria II flow cytometer (BD Biosciences); Precision epitope mapping (Pepscan Presto BV, Lelystad, The Netherlands).
Data analysis	Octet BLI software (Pall ForteBio, version 10.0); PRISM software (GraphPad, version 9); Excel (Microsoft, version 2012); AmyloFit open source software (https://www.amylofit.ch.cam.ac.uk,); Image J/FiJi software (https://imagej.nih.gov/ij, version 1.0); Image-Pro Premier software (Media Cybernetics, version 9.3); BD FACS Diva software (BD Biosciences, version 6.1.3); FlowJo software (Tree Star Inc., version 9).

For manuscripts utilizing custom algorithms or software that are central to the research but not yet described in published literature, software must be made available to editors and reviewers. We strongly encourage code deposition in a community repository (e.g. GitHub). See the Nature Portfolio guidelines for submitting code & software for further information.

Data

Policy information about availability of data

All manuscripts must include a data availability statement. This statement should provide the following information, where applicable:

- Accession codes, unique identifiers, or web links for publicly available datasets
- A description of any restrictions on data availability
- For clinical datasets or third party data, please ensure that the statement adheres to our policy

The main data supporting the results in this study are available within the paper and its supplementary information. Source data are provided with this paper. Source data are provided with this paper.

Research involving human participants, their data, or biological material

Policy information about studies with human participants or human data. See also policy information about sex, gender (identity/presentation), and sexual orientation and race, ethnicity and racism.

Reporting on sex and gender	Isolated islets form both male and female sexes were considered in the study and analysis. No disseagregated sex-based post-hoc analysis was performed due to low sample size.
Reporting on race, ethnicity, or other socially relevant groupings	De-identified blood samples for antibody screening were obtained from healthy elderly donors. No data on ethnicity were available from the human islet donors.
Population characteristics	Healthy elderly donors ≥ 60 years were included for antibody screening.<br>Donor characteristics for human donors providing islets are reported in Supplementary Table 2.
Recruitment	Healthy elderly blood donors were recruited through local advertisement. There are no known biases that are likely to impact the results of this manuscript.
Ethics oversight	Human blood donation was approved by Ethics Committee of the Canton of Zürich under written informed consent from the blood donors. For human islet studies, donors or their families provided written informed consent and approval was obtained from the Human Research Ethics Board of the University of Alberta and the Institutional Ethical Committee of the University and the University Hospital of Lille.

Note that full information on the approval of the study protocol must also be provided in the manuscript.

Field-specific reporting

Please select the one below that is the best fit for your research. If you are not sure, read the appropriate sections before making your selection.

[x] Life sciences   [ ] Behavioural & social sciences   [ ] Ecological, evolutionary & environmental sciences

For a reference copy of the document with all sections, see nature.com/documents/nr-reporting-summary-flat.pdf

Life sciences study design

All studies must disclose on these points even when the disclosure is negative.

Sample size	No sample size calculations were performed. The sample sizes were determined based on previous literature (Butler AE, et al., Diabetes. 2004; Matveyenko AV, et al., Diabetes, 1996; Couce M, et al. Diabetes, 1996) allowing for statistical analyses.
Data exclusions	For in vitro studies no data were excluded. For in vivo studies animals were excluded due to 1) misgenotyping or 2) premature death.
Replication	All main effects reported in this study were observed repeatedly using identical or similar study designs. For most in vitro assays 3 independent experiments were performed. In vivo efficacy was replicated in 4 independent studies with similar settings. The exact number of replicates is indicated in the figure legends.
Randomization	For immunization, transgenic rats and mice were randomized and allocated to the different treatment groups based on body weight and blood glucose concentration during an initial oral glucose tolerance test. Human islet-engrafted mice were allocated to the treatment groups according to blood glucose levels prior transplant (NSG mice) and two or eight weeks post-transplant (Rag2 null mice).<br>For all other experiments, treatments were randomly allocated.
Blinding	For cell-based assays and experiments involving human islets investigators were blinded to treatment group allocation during data collection and data analysis until full study completion. For in vivo studies, investigators were fully blinded to treatment group allocation upon randomization until full completion of in vivo phase and subsequent tissue and biofluid collection and analysis.

Reporting for specific materials, systems and methods

We require information from authors about some types of materials, experimental systems and methods used in many studies. Here, indicate whether each material, system or method listed is relevant to your study. If you are not sure if a list item applies to your research, read the appropriate section before selecting a response.

Materials & experimental systems

n/a	Involved in the study
	x Antibodies
	x Eukaryotic cell lines
x	Palaeontology and archaeology
	x Animals and other organisms
x	Clinical data
x	Dual use research of concern
x	Plants

Methods

n/a	Involved in the study
x	ChIP-seq
	x Flow cytometry
x	MRI-based neuroimaging

Antibodies

Antibodies used

Primary antibodies:
α-IAPP-O and variants, Neurimmune proprietary reagent
human IgG isotype control, Neurimmune proprietary reagent
mouse chimeric α-IAPP-O and variants, Neurimmune proprietary reagent
rat chimeric α-IAPP-O and variants, Neurimmune proprietary reagent
anti-idiotypic human Fab antibody fragment (custom-made, 1 μg/ml, AbD Serotec)
rabbit anti-IAPP (α-IAPP; T-4145, 2 μg/ml, Peninsula Laboratories International),
rabbit anti-IAPP (α-IAPP; T-4157, 1:500, Peninsula Laboratories International)
mouse monoclonal anti-IAPP (R10/99, 1:100, ab115766, Abcam)
mouse monoclonal anti-human IAPP (E-5, 1:100, sc-377530, Santa Cruz Biotechnology)
guinea pig polyclonal anti-insulin (IR002, 1:3, DAKO)
mouse monoclonal anti-rat CD68 (MCA341GA, 1:1000, Bio-Rad)
rat monoclonal anti-mouse CD68 (ab53444, 1:200, Abcam)
mouse anti-Aβ-amyloid 6E10 (30 nM, BioLegend)
rabbit anti-IL-1β (ab9722, 1:2500, Abcam)
mouse anti-β-actin (A1978, 1:100000, Sigma)

Secondary antibodies:
HRP-conjugated donkey anti-human antibody, Jackson ImmunoResearch, #709-036-098, 1:10000 (ImmunoBlots); 1:2000 (ELISA)
HRP-conjugated goat anti-rabbit antibody, Jackson ImmunoResearch, #111-035-045, 1:10000
HRP-conjugated goat anti-rabbit antibody, Jackson ImmunoResearch, #111-035-144, 1:10000
HRP-conjugated goat anti-mouse antibody, Jackson ImmunoResearch, #115-035-003, 1:10000
HRP-conjugated goat anti-mouse antibody, Jackson ImmunoResearch, #115-035-146, 1:10000
HRP-conjugated mouse anti-rat IgG2b antibody, SouthernBiotech, #3070-05, 1:5000
HRP-conjugated mouse anti-rat kappa antibody, SouthernBiotech, #3090-05, 1:5000
HRP-conjugated rabbit anti-FITC antibody, Origene, #BP464HRP, 1:30000
Cy2-conjugated donkey anti-human antibody, Jackson ImmunoResearch, #709-225-149, 1:250
Cy2-conjugated donkey anti-rabbit antibody, Jackson ImmunoResearch, #711-225-152, 1:100
Cy3-conjugated mouse anti-rat antibody, Jackson ImmunoResearch, #212-165-082, 1:200
Cy3-conjugated donkey anti-mouse antibody, Jackson ImmunoResearch, #715-165-150, 1:200
TRITC-conjugated goat anti-guinea pig antibody, 1:200; Jackson ImmunoResearch, #106-025-003, 1:200
Cy5-conjugated donkey anti-human antibody, Jackson ImmunoResearch, #709-175-149, 1:200
Cy5-conjugated donkey anti-mouse antibody, Jackson ImmunoResearch, #715-175-150, 1:200
biotinylated donkey anti-mouse antibody, Jackson ImmunoResearch, #715-065-150, 1:500

Validation

Primary Antibodies have been validated for use in immunofluorescence/immuno-blotting by the manufacturers as stated on their respective websites as well in previous publications cited below:

α-IAPP-O and variants, IgG control and anti-idiotypic human Fab: data provided in the manuscript.
Rabbit anti-IAPP (T-4145): Matveyenko et al., 2009 (ref. 26 in the paper).
Rabbit anti-IAPP (T-4157): Rivera et al., 2014 (ref. 57 in the paper).
Mouse monoclonal anti-IAPP (R10/99): https://www.abcam.com/amylindap-antibody-r1099-ab115766.html.
Mouse monoclonal anti-human IAPP (E-5): Zou et al., 2019 (ref. 18 in the paper).
Guinea pig polyclonal anti-insulin: Abedini et al., 2016 (ref. 22 in the paper); Westwell-Roper et al., 2014 (ref. 27 in the paper); Potter et al., 2010 (ref. 48 in the paper).
Mouse monoclonal anti-rat CD68: https://www.bio-rad-antibodies.com/monoclonal/rat-cd68-antibody-ed1-mca341.html?f=purified.
Rat monoclonal anti-mouse CD68: https://www.abcam.com/cd68-antibody-fa-11-ab53444.html.
Mouse anti-Aβ-amyloid 6E10: Sevigny et al., 2016 (ref. 84 in the paper).

Rabbit anti-IL-1β: https://www.abcam.com/il-1-beta-antibody-ab9722.html.
Mouse anti-β-actin: Westwell-Roper et al., 2014 (ref. 27 in the paper).

Eukaryotic cell lines

Policy information about cell lines and Sex and Gender in Research

Cell line source(s)	Rat insulinoma INS-1 beta cells (INS-1 832/13, Cat# SCC207, Sigma), human THP-1 monocytes (Cat# TIB-202, ATCC).
Authentication	None of the cell lines used were authenticated.
Mycoplasma contamination	Cell lines were not tested for mycoplasma contamination.
Commonly misidentified lines (See ICLAC register)	No commonly misidentified cell lines used in this study.

Animals and other research organisms

Policy information about studies involving animals; ARRIVE guidelines recommended for reporting animal research, and Sex and Gender in Research

Laboratory animals	Two months old hemizygous hIAPP transgenic male rats (RIP-HAT; CD:SD-Tg(ins2-IAPP)Soel) and wild-type male Sprague-Dawley (SD) rats were obtained from Charles River Laboratories (Germany) and housed under controlled conditions (22±2ºC, 12:12 hour light/dark cycle with light phase from 2:00 am to 2:00 pm and 40-60% humidity) with free access to standard chow diet (Extrudate 3436, KLIBA NAFAG, Switzerland) and water.<br><br>NSG male mice (NOD.Cg-Prkdcscid Il2rgtm1Wjl/Sz, #005557, The Jackson Laboratory, USA) aged 6 to 20 weeks, and Rag2 null male mice (B6.129S6-Rag2tm1Fwa N12, #RAGN12-M, Taconic Biosciences, Lille Skensved, DK) aged 13 to 57 weeks and placed on a high-fat diet (HFD, NJ D12450B, Research Diets, New Brunswick) two weeks post-transplant were used for human islet transplantation studies. NSG mice were maintained as follows: 14:10 hour light/dark cycle, 22±1 ºC and 50-60% humidity on chow diet (6% fat, Teklab 2918, Huntingdon, UK). RAG2 null mice were kept as follows: 12:12 hour light/dark cycle, 20-24ºC and 40-60% humidity on chow diet (LFD, NJ D12450B, Research Diets, New Brunswick) until two week post-transplant.<br><br>8-40 week old hemizygous hIAPP transgenic male mice (FVB/N-Tg(Ins2-IAPP)RHFSoel/J, #008232, The Jackson Laboratory, USA) were bred with 8-40 week old DBA/2J wild-type female mice (#000671, The Jackson Laboratory, USA) to generate hIAPP transgenic and wild-type F1 male mice on a mixed FVB/N and DBA/2J background that were housed under controlled conditions (22±2ºC, 12:12 hour light/dark cycle with light phase from 2:00 am to 2:00 pm and and 40-60% humidity) with free access to standard chow diet (Extrudate 3436, KLIBA NAFAG, Switzerland) and water.
Wild animals	The study did not involve wild animals.
Reporting on sex	Findings in transgenic animals apply to male mice only as no female animals have been tested.
Field-collected samples	The study did not involve samples collected from the field.
Ethics oversight	All animal handling, welfare, monitoring, and euthanasia practices were performed in strict accordance with the ethical guidelines. Experiments involving transgenic and wild-type rats and mice were approved by the Veterinary Office of the Canton Zurich, Switzerland (authorization numbers 143/2015 and 150/2012) and performed as recommended by the Swiss Federal Veterinary Office (FVO). Studies on human islet-engrafted mice were approved by the Animal Care Committee and the Clinical Research Ethics Board of the University of British Columbia (NSG mice), and by the institutional ethical committee of the University of Lille and the Centre Hospitalier Régional Universitaire de Lille (Rag2 null mice).

Note that full information on the approval of the study protocol must also be provided in the manuscript.

Flow Cytometry

Plots

Confirm that:

- [x] The axis labels state the marker and fluorochrome used (e.g. CD4-FITC).
- [x] The axis scales are clearly visible. Include numbers along axes only for bottom left plot of group (a 'group' is an analysis of identical markers).
- [x] All plots are contour plots with outliers or pseudocolor plots.
- [x] A numerical value for number of cells or percentage (with statistics) is provided.

Methodology

Sample preparation	Peripheral blood mononuclear cells (PBMCs) were enriched from healthy donor blood using monocyte isolation kit (Miltenyi Biotec) and differentiated into macrophages in serum-free medium (M-SFM, ThermoFisher Scientific) supplemented with 100

ng/mL GM-CSF (Gibco) and 100 µg/ml penicillin/streptomycin for 6 days at 37°C and 5% CO_2. PBMC-derived macrophages were plated (5 x 10 to the power of 5 cells/well) and cultured in M-SFM medium supplemented with 100 µg/ml penicillin/streptomycin, 100 ng/mL GM-CSF, 1 ng/mL LPS (Sigma) and 20 ng/mL IFN-γ one day prior to the experiment. PBMC-derived macrophages were incubated with a 1:10 dilution of the solution containing pHrodo-labeled hIAPP aggregates (20 µM) and antibodies (0, 1.5, 3, 6 and 12 nM) in fresh M-SFM medium supplemented with 100 µg/ml penicillin/streptomycin and 50 µg/mL of the scavenger receptor inhibitor Fucoidan (F5631, Sigma) for 30min at 37°C. Human Fc receptor (FcR) blocking solution (1:10 dilution; 130-059-901, Miltenyi Biotec) and cytochalasin D (50 µg/ml; C2618, Sigma) were added to inhibit FcR-dependent and general phagocytosis. After detachment, macrophages were washed in PBS and fluorescence of any surface-bound pHrodo-labeled hIAPP was quenched by addition of trypan blue (10%).
Human THP-1 cells were seeded at a density of 5 x 10 to the power of 5 cells/ml and differentiated in RPMI-1640 medium (30-2001, ATCC) supplemented with 100 µg/ml penicillin/streptomycin, 10% FBS and 25 ng/mL PMA (Sigma) for 72h at 37°C and 5% CO_2. IFN-γ (20 ng/mL) was added to the culture medium for the last 24 hours. pHrodo-labeled hIAPP and antibodies were added to THP-1 macrophages plated in fresh RPMI-1640 medium supplemented with 100 µg/ml penicillin/streptomycin and 50 µg/mL of the scavenger receptor inhibitor Fucoidan (F5631, Sigma) for 1h at 37°C. THP-1 macrophages were detached and washed in PBS and fluorescence of any surface-bound pHrodo-labeled hIAPP was quenched by addition of trypan blue (10%).
Intracellular pHrodo green was excited using a 488 nm laser and the fluorescence emission was collected using a 530/30 nm filter (FITC).

Instrument	FACS Aria II flow cytometer equipped with BD FACS Diva software (BD Biosciences)
Software	Data were exported as Flow Cytometry Standard format 3.0 files (FCS files) and analyzed with FlowJo software (Tree Star Inc.).
Cell population abundance	A minimum of 10’000 events were acquired from each sample.
Gating strategy	Gating was done on single macrophages with high forward and side scatter (FSC-A and SSC-A) levels, and pHrodo-hIAPP-positive macrophages with fluorescence emission above cytochalasin D-treated macrophages (negative control) were counted.

☐ Tick this box to confirm that a figure exemplifying the gating strategy is provided in the Supplementary Information.
